# Underestimated health risks: polystyrene micro- and nanoplastics jointly induce intestinal barrier dysfunction by ROS-mediated epithelial cell apoptosis

**DOI:** 10.1186/s12989-021-00414-1

**Published:** 2021-06-07

**Authors:** Boxuan Liang, Yizhou Zhong, Yuji Huang, Xi Lin, Jun Liu, Li Lin, Manjiang Hu, Junying Jiang, Mingzhu Dai, Bo Wang, Bingli Zhang, Hao Meng, Jesse Justin J. Lelaka, Haixia Sui, Xingfen Yang, Zhenlie Huang

**Affiliations:** 1 Department of Toxicology, Guangdong Provincial Key Laboratory of Tropical Disease Research, School of Public Health, Southern Medical University, 1023-1063 Shatai Nan Road, Guangzhou, 510515 PR China; 2 Faculty of Preventive Medicine, School of Public Health, Guangdong Pharmaceutical University, Guangzhou, 510006 PR China; 3 Hunter Biotechnology, Inc., Hangzhou, 310051 PR China; 4 Division III of risk assessment, China National Center for Food Safety Risk Assessment, Beijing, 100022 PR China; 5 Food Safety and Health Research Center, School of Public Health, Southern Medical University, 1023-1063 Shatai Nan Road, Guangzhou, 510515 PR China

**Keywords:** Microplastic, Nanoplastic, Mixture, Intestinal barrier, Health risk, Combined effect

## Abstract

**Background:**

Micro- and nanoplastic pollution has become a global environmental problem. Nanoplastics in the environment are still hard to detect because of analysis technology limitations. It is believed that when microplastics are found in the environment, more undetected nanoplastics are around. The current “microplastic exposure” is in fact the mixture of micro- and nanoplastic exposures. Therefore, the biological interaction between organisms among different sizes of micro- and nanoplastics should not be neglected.

**Results:**

We measured the biodistribution of three polystyrene (PS) particles (50 nm PS, PS50; 500 nm PS, PS500; 5000 nm PS, PS5000) under single and co-exposure conditions in mice. We explored the underlying mechanisms by investigating the effects on three major components of the intestinal barrier (the mucus layer, tight junctions and the epithelial cells) in four intestine segments (duodenum, jejunum, ileum and colon) of mice. We found that the amounts of both PS500 and PS5000 increased when they were co-exposed with PS50 for 24 h in the mice. These increased amounts were due primarily to the increased permeability in the mouse intestines. We also confirmed there was a combined toxicity of PS50 and PS500 in the mouse intestines. This manifested as the mixture of PS50 and PS500 causing more severe dysfunction of the intestinal barrier than that caused by PS50 or PS500 alone. We found that the combined toxicity of PS micro- and nanoplastics on intestinal barrier dysfunction was caused primarily by reactive oxygen species (ROS)-mediated epithelial cell apoptosis in the mice. These findings were further confirmed by an oxidants or antioxidants pretreatment study. In addition, the combined toxicity of PS micro- and nanoplastics was also found in the mice after a 28-day repeated dose exposure.

**Conclusions:**

There is a combined toxicity of PS50 and PS500 in the mouse intestines, which was caused primarily by ROS-mediated epithelial cell apoptosis in the mice. Considering that most recent studies on PS micro- and nanoplastics have been conducted using a single particle size, the health risks of exposure to PS micro- and nanoplastics on organisms may be underestimated.

**Supplementary Information:**

The online version contains supplementary material available at 10.1186/s12989-021-00414-1.

## Background

Plastic pollution has become a global problem, in both land and marine environments [1]. Waste plastics are continuously degraded by various physicochemical processes, generating so-called microplastics (size < 5 mm) and nanoplastics (size < 100 nm), based upon the diameter of the plastic particles [2]. Due to the small plastic particle size, micro- and nanoplastics are easily ingested by organisms and enter their bodies where they affect biological health [3, 4]. Recent data have revealed that micro- and nanoplastics are found in aquatic foods, tap water, beer, sea salt and packaged beverages [5–8]. These compounds have also been detected in human stool [9]. Given that multiple toxicities of micro- and nanoplastics have been found in both aquatic and terrestrial organisms, all humans have a degree of exposure to micro- and nanoplastics [10–14].

Particle size is one of the most important factors in determining the extent and pathway of particle biodistribution [15, 16], affecting the particle’s toxicities in vivo [17, 18]. The upper limit for microplastic particles to translocate across the human gut is about 150 μm [19]. However, particles > 1.5 μm rarely penetrate into the organs, and thus are unlikely to cause any organ damage [15]. The absorption of micro- and nanoplastics increases in organisms as particle size decreases [20–23]. Despite what is known, more research is needed on the adverse effects of microplastics and nanoplastics on human health.

Waste plastic particles occur in an array of sizes, shapes and materials [8, 24–26]. However, nanoplastics in the environment are still hard to detect because of analysis technology limitations [27]. It is estimated that the particle count will scale inversely with the particle radius to the power of 3 [28]. Additionally, it is believed that when microplastics are found in the environment, more undetected nanoplastics are around as well [29]. The current “microplastic exposure” is in fact the mixture of micro- and nanoplastic exposure [30, 31]. An in vitro study has reported that a mixture of different sizes of nanoparticles affected their cellular uptake efficiency [32]. This promotes the hypothesis that there is also interplay between the sizes of micro- and nanoplastics. This affects their biodistribution and toxicities in vitro and in vivo. However, most studies on micro- and nanoplastics have used a single size of plastic particles in the exposure group so far [33]. The biological interaction between different sizes of micro- and nanoplastics on organisms may be neglected [34]. This gives rise to the need for additional, foundational studies to understand the underlying mechanisms of interaction.

In the present study, we explored the interplay between polystyrene (PS) micro- and nanoplastic size, and how it influences their biodistribution and toxicity. We measured the biodistribution of three PS particles (50 nm PS, PS50; 500 nm PS, PS500; 5000 nm PS, PS5000) under single and co-exposure conditions in mice. We explored the underlying mechanisms by investigating the effects on three major components of the intestinal barrier (the mucus layer, tight junctions and epithelial cells) in four mouse intestine segments (duodenum, jejunum, ileum and colon). To confirm the combined toxicity of PS micro- and nanoplastics in the intestine, we also conducted a 28-day repeated dose oral toxicity study in mice using a range of doses (2.5–500 mg/kg body weight).

## Results

### Micro- and nanoplastic characteristics

**Fig. 1:**
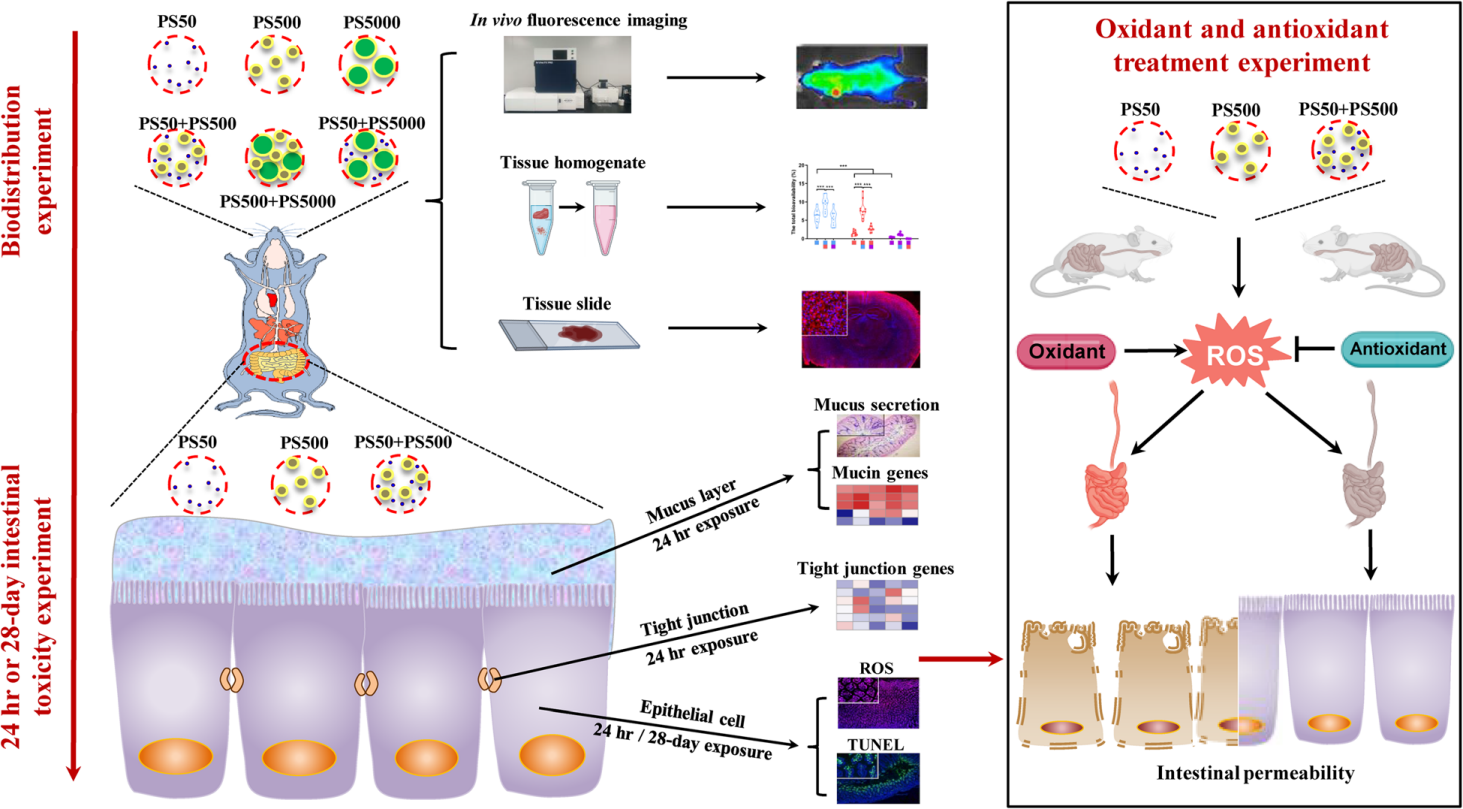
Flow chart diagram for the present study. We used D-galactose (1000 mg/kg body weight, dissolved in H_2_O) and lipopolysaccharide (10 mg/kg body weight, dissolved in H_2_O) as oxidants; lipoic acid (100 mg/kg body weight, suspended in 1% carboxymethylcellulose) and melatonin (40 mg/kg body weight, suspended in 1% carboxymethylcellulose) as antioxidants. ROS, reactive oxygen species

**Fig. 2:**
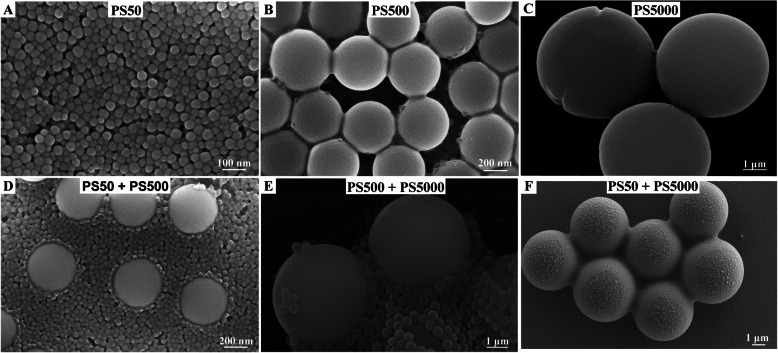
Characterization of the NF-PS50, NF-PS500 and NF-PS5000. Representative SEM images of **A**) PS50, **B**) PS500, **C**) PS5000, **D**) mixture of PS50 and PS500, **E**) mixture of PS500 and PS5000 and **F**) mixture of PS50 and PS5000. NF, non-fluorescence

We have presented the present study’s strategy with a flow chart diagram (Fig. 1). Scanning electron microscope (SEM) results showed that all non-fluorescence (NF) PS particles were spherical. The mixture of two particle sizes did not change the particle morphology in water (Fig. 2). SEM images showed that the average sizes were 50.7, 503.6, and 5047.0 nm for the PS50, PS500, and PS5000, respectively. The characteristics of PS50, PS500 and a mixture of PS50 and PS500 in double distilled water, as well as simulated gastric and intestinal fluids were measured by Zetasizer Nano ZS. This included hydrodynamic size, polymer dispersity index (PDI), and zeta potential. We found that both PS50 and PS500 had narrow size distributions and good stabilities in double distilled water. The high zeta potential indicated that the mixture of PS50 and PS500 was monodisperse in double distilled water. PS50 and PS500 were aggregated in simulated gastric and intestinal fluids, manifesting as hydrodynamic sizes. PDI increased and zeta potentials decreased. The physical characteristics of red fluorescence (RF) and green fluorescence (GF) particles are similar to those of NF particles (Table S1). We also confirmed that the fluorescence leakage from each particle type was negligible under the experimental conditions (Figure S1).

### PS micro- and nanoplastics were distributed in a size-dependent manner in the mice

The dynamic biodistribution of different sized PS particles in the mice under single exposure was monitored at 1, 6, 12 and 24 h after exposure by the in vivo fluorescence imaging system (Figure S2). We found that the fluorescent PS particles moved over time and accumulated in the intestine during 24 h exposure. However, the in vivo fluorescence imaging system detected no other fluorescent signals in any other organs.

**Fig. 3:**
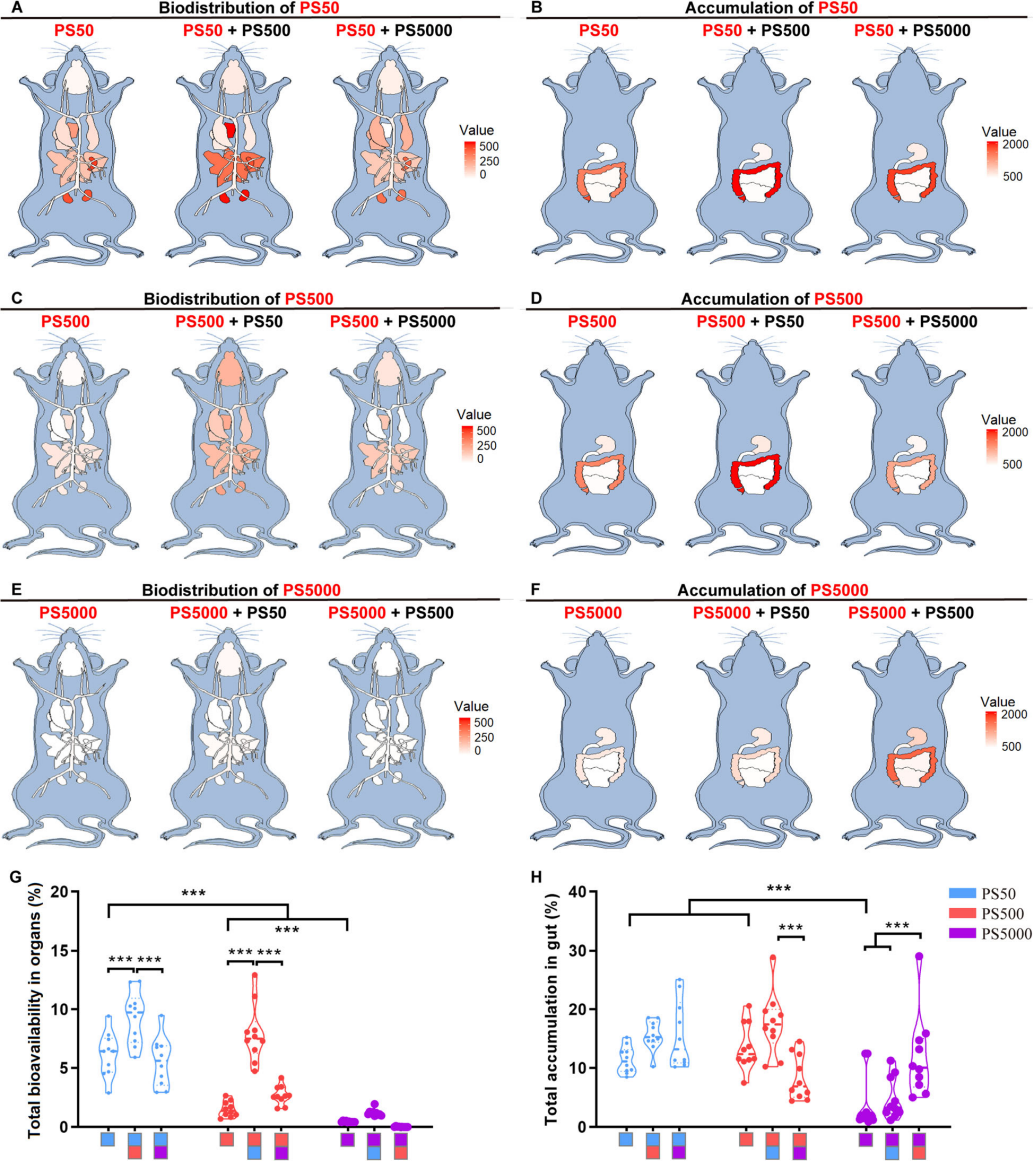
Biodistribution of PS50, PS500 and PS5000 in the mice after 24 h exposure. Summarize diagrams showed the **A**) organ biodistribution and **B**) intestinal accumulation of PS50 after a single exposure of PS50 or co-exposure with PS500 or PS5000 for 24 h. Summarize diagrams showed the **C**) organ biodistribution and **D**) intestinal accumulation of PS500 after a single exposure of PS500 or co-exposure with PS50 or PS5000 for 24 h. Summarize diagrams showed the **E**) organ biodistribution and **F**) intestinal accumulation of PS5000 after a single exposure of PS5000 or co-exposure with PS50 or PS500 for 24 h. **G** Total bioavailability of PS particles. **H** Total accumulation of PS particles in the intestine. ***P < 0.001, comparisons were made with ANOVA, followed by Tukey’s method (*n* = 10 per group). The color bars were used for grouping, one bar represented a dose of 250 mg/kg body weight, and two bars with different color represented a mix exposure with a dose of 250 mg/kg body weight of each particle

The micro- and nanoplastic biodistribution and the accumulation in the intestine and other organs under single exposure were further examined after homogenizing each organ. The standard curve for each organ and for the blood showed that the fluorescence intensity of particles of each size was linearly correlated with their concentrations (Figure S3). The biodistribution of the PS micro- and nanoplastics in other organs and the accumulation in the intestine are summarized in the mouse model diagrams (Fig. 3A-F). In general, the PS particles were distributed in other organs in a size-dependent manner after 24 h exposure, in which the smaller the particle size, the more biodistribution there was. The total bioavailability for PS50, PS500 and PS5000 were 6.16, 1.53 and 0.46%, respectively (Figs. 3G and S4A). PS50 and PS500 were found in the spleen, kidneys, heart, liver, lungs, blood, testis and epididymis, brain and thighbone, in the order of the PS concentration in each organ (Figure S4C-H, J, L, N). Moreover, there were some individual signs of PS50 and PS500 in the muscles and breastbone (Figure S4K, M) and some PS50 in the ovaries and uterus (Figure S4I). As for the PS5000, it was found in the blood, but not in any other organs (Figure S4L). No difference by sex was observed in regards to the organ biodistribution of these three PS sizes, with the exception of PS500 in the spleen (Figure S4G). The total accumulations of PS50, PS500 and PS5000 in the intestine were 11.41, 13.66 and 3.84%, respectively (Figs. 3H and S4B). The accumulation of PS50 and PS500 between the small and large intestine was similar, yet the accumulation of PS50 was lower than that of PS500 in the stomach (Figure S4O-Q). Compared with the accumulation in the stomach and small intestine, a greater percentage of the PS50 and PS500 accumulated in the large intestine. The PS5000 accumulated primarily in the stomach and large intestine (Figure S4O-Q).

### Micro- and nanoplastic co-exposure facilitated both particles’ biodistribution in the mice

To explore whether there is any interplay between PS particles of different sizes, each of the two sizes of PS particles were mixed with the same concentration in the biodistribution experiments. Mice were then exposed to the mixtures. The dynamic biodistribution of different size PS particles in the mice under co-exposure conditions was monitored at 1, 6, 12 and 24 h after exposure (Figure S5). We found that the two PS particles moved simultaneously to the intestine over time in the PS50 + PS500 group, whereas the two PS particles moved independently to the intestine over time in the PS500 + PS5000 and PS50 + PS5000 groups (Figure S5). The total PS50 bioavailability increased when it was co-exposed with PS500, compared with the PS50 exposure alone (Fig. 3G). The PS50 biodistribution increased in the heart, blood and thighbone when it was co-exposed with PS500, compared with the PS50 exposure alone (Figure S6B, J, L). Meanwhile, the total PS500 bioavailability increased when it was co-exposed with PS50, compared with the PS500 exposure alone (Fig. 3G). The PS500 biodistribution increased in the brain, heart, lungs, spleen, kidneys, testis and epididymis, blood and breastbone when it was co-exposed to PS50, compared with the PS500 exposure alone (Figure S6A, B, C, E, F, H, J, K). Additionally, the PS5000 biodistribution increased in the brain and blood when co-exposed with PS50, compared with the PS5000 exposure alone (Figure S6A, J). Of note, the biodistribution of PS500 and PS5000 in the blood dropped when these two PS particles were mixed (Figure S6J). In addition, co-exposure of PS50 and PS500 did not increase the biodistribution of these two PS particles in the liver, ovaries or uterus and muscle tissue, compared with PS50 or PS500 exposure alone (Figure S6D, G, I). Co-exposure to the other two PS sizes did not affect the total accumulation of PS50 or PS500 in the stomach, or in the small intestine (Figs. 3H and S6M, N), but the PS500 accumulation increased in the large intestine when it was mixed with PS50, compared with the single exposure group (Figure S6O). In particular, the total PS5000 accumulation in the intestine increased when it was co-exposed to PS500, compared with the single exposure group and the PS50 + PS5000 group (Fig. 3H).

To confirm the interplay between PS50 and PS500 on the biodistribution and accumulation in vivo, we changed the concentration of one of the PS particles, while maintaining the concentration of the other PS particles. The organ biodistribution results showed that PS50’s total bioavailability increased as PS500 concentration increased (Figure S7A), accompanied with biodistribution increased in the heart, blood and thighbone (Figure S7D, L, N). We also found that the PS50 biodistribution in the brain increased when it was co-exposed with two-fold of PS500 (Figure S7C). Similarly, the PS500’s total bioavailability and the biodistribution in the brain, heart, lungs, spleen, kidneys, testis and epididymis, and blood increased as the PS50 concentration increased (Figure S7A, C-E, G, H, J, L). Co-exposure with PS50 and PS500 did not change the biodistribution of these two PS particles in the liver, ovaries and uterus, muscle, breastbone or small intestine, compared with PS50 or PS500 exposure alone (Figure S7F, I, K, M, P). It was found that the PS50 accumulation in the stomach increased as the concentration of PS500 increased (Figure S7O). The PS500 accumulation in the large intestine also increased as the PS50 concentration increased (Figure S7Q). However, co-exposure to PS50 and PS500 did not increase the total accumulation of these two PS particles in the intestine, compared with PS50 or PS500 exposure alone (Figure S7B).

### Histopathology confirmed the biodistribution of PS micro- and nanoplastics in the organs of mice

**Fig. 4:**
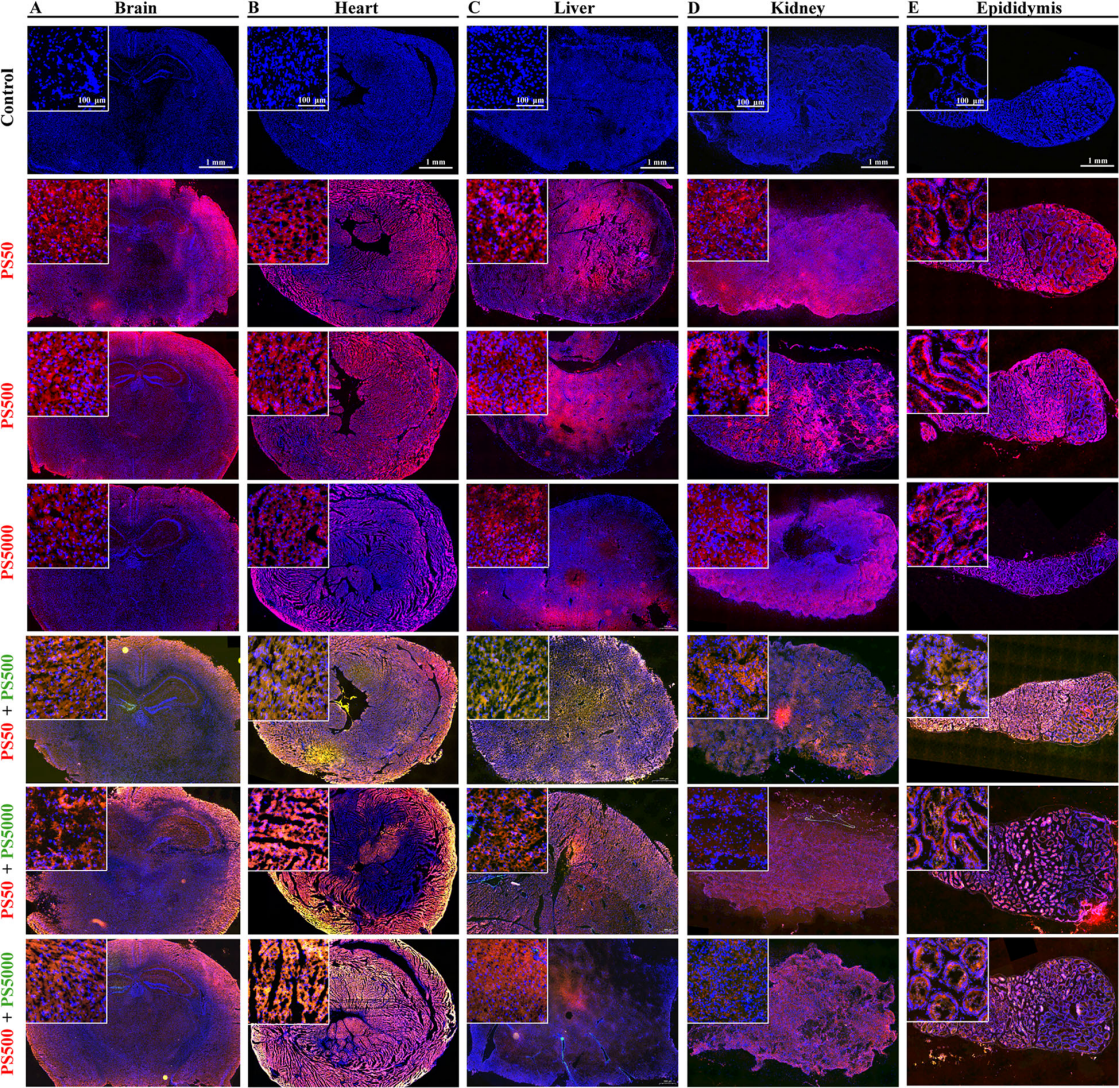
Histopathology confirmed the biodistribution of PS micro- and nanoplastics in the organs. Representative merged fluorescence images showed the biodistribution of PS50, PS500, PS5000 and their mixtures in **A**) brain, **B**) heart, **C**) liver, **D**) kidney and **E**) epididymis after 24 h exposure (*n* = 5 per group). The font color represented the fluorescent color of the particle. The nucleus was stained with DAPI in blue

The biodistribution of PS micro- and nanoplastics in each mouse organ was confirmed by fluorescence histopathology. The results showed that these three sizes of PS particles were found in the brain, heart, liver, lungs, spleen, kidneys, testis and epididymis (Figs. 4 and S8). The biodistribution of PS50 and PS500 in all of the organs was exceeded by that of PS5000 (Figs. 4 and S8). The PS particles were microscopically observed in the brain, heart, liver, kidneys and testis (Figs. 4A-D and S8C), and they were also found in the blood vessels of the lungs and spleen (Figure S8A, B). The PS particles were predominantly found in the lumen of the epididymis (Fig. 4E). The biodistribution of PS50 and PS500 increased in the mouse organs when these two PS particles were co-exposed (Figs. 4 and S8). Of note, PS50 and PS500 were more distributed in the organs than PS5000, when co-exposed with PS5000 (Figs. 4 and S8).

### The combined effects of PS micro- and nanoplastics aggravated intestinal barrier dysfunction in the mice

**Fig. 5:**
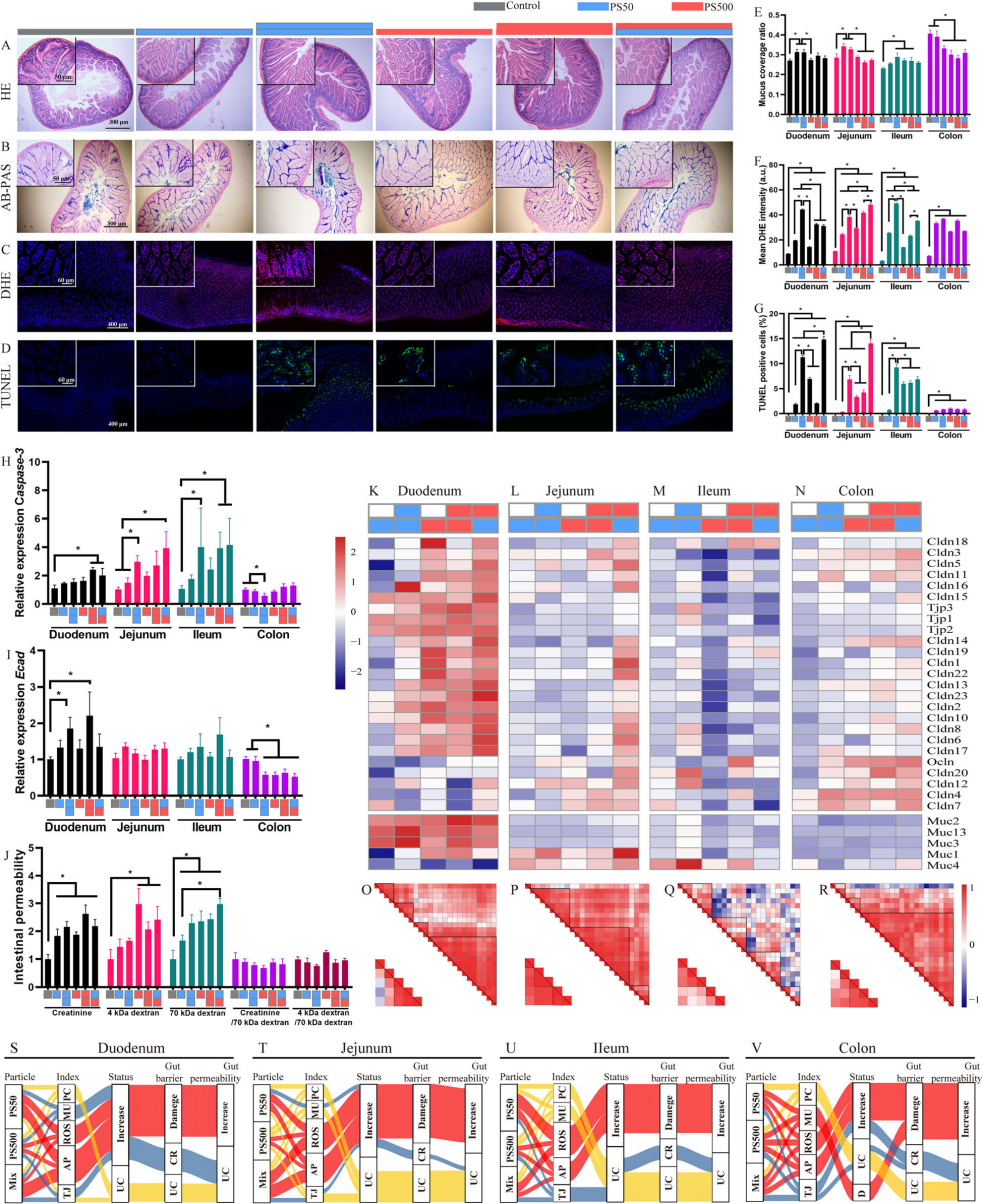
Mixture of PS micro- and nanoplastics aggravated dysfunction of intestinal barrier in mice. Representative merged images of the jejunum sections **A**) stained with H&E to assess the pathological changes; **B** stained with AB-PAS to assess the change of mucin secretion, the mucin was stained in blue; **C** stained with DHE to assess the ROS generation, the ROS was stained in red and the nucleus was stained with DAPI in blue; **D** stained with TUNEL to assess cell apoptosis, the apoptosis cells were stained in green and the nucleus was stained with DAPI in blue. The quantification of **E**) mucus coverage ratio, **F**) mean DHE intensity and **G**) TUNEL positive cell rate in the four intestinal segments. The mRNA expression of H) *Casepase-3* and **I**) *Ecad* in the four intestinal segments were shown. **J** The fluxes of creatinine, 4 kDa dextran, 70 kDa dextran in the intestine were quantified. The mRNA expressions of TJPs and mucins genes were shown in the **K**) duodenum, **L**) jejunum, **M**) ileum and **N**) colon. The correlation of TJPs genes expression and mucins genes expression in the **O**) duodenum, **P**) jejunum, **Q**) ileum and **R**) colon. Sankey diagrams summarized multiple effects of PS particles in the **S**) duodenum, **T**) jejunum, **U**) ileum and **V**) colon. *P < 0.05. Results are shown as means ± SE. Comparisons were made with ANOVA, followed by Tukey’s method (*n* = 5 per group). The color bars were used for grouping, one bar represented a dose of 250 mg/kg body weight, two bars with same color represented a dose of 500 mg/kg body weight, and two bars with different colors represented the mix exposure group with a dose of 250 mg/kg body weight of each particle. PC, pathological change; MU, mucin secretion; AP, apoptosis; TJ, tight junction; UC, unchanged; CR, compensatory response

To elucidate the effects of PS micro- and nanoplastics exposure on the intestinal barrier, we performed hematoxylin-eosin (H&E) staining, alcian blue-periodic acid schiff (AB-PAS) staining, dihydroethidium (DHE) staining, TdT-mediated dUTP Nick-End Labeling (TUNEL) staining and mRNA detection of tight junction proteins (TJPs) and mucin genes in the duodenum, jejunum, ileum and colon. H&E staining found no observable pathological change in the four intestinal segments (Figs. 5A and S9A, E, I). AB-PAS staining showed that mucus secretion increased after exposure to PS50 in the duodenum, jejunum and ileum, whereas it decreased after exposure to PS50 and PS500 in the colon (Figs. 5B, E and S9B, F, J). Similarly, the mRNA mucin gene levels increased in the duodenum, jejunum and ileum, whereas they decreased in the colon (lower panel of Fig. 5K-N). DHE staining revealed that both PS50 and PS500 induced significant ROS generation in the four intestinal segments in a dose-dependent manner (Figs. 5C and S9C, G, K). PS50 induced more ROS generation than PS500 in the duodenum and ileum. The mixture of PS50 and PS500 induced more ROS generation than PS50 or PS500 alone in the jejunum (Fig. 5F). Meanwhile, TUNEL staining showed that both PS50 and PS500 induced epithelial cell apoptosis in a dose-dependent manner in the duodenum, jejunum and ileum (Figs. 5D and S9D, H, L). PS50 induced more serious epithelial cell apoptosis than PS500 in the duodenum, jejunum and ileum (Fig. 5G). The mixture of PS50 and PS500 induced more serious epithelial cell apoptosis than PS50 or PS500 alone in the duodenum and jejunum (Fig. 5G). The increased *caspase-3* mRNA level in the duodenum, jejunum and ileum validated the increased apoptosis detected by TUNEL staining (Fig. 5H).

The TJP genes’ mRNA, including *claudin* (*Cldn*) and *zonula occludens* (*ZO*), was evaluated by qPCR and normalized to the epithelial cell biomarker *E-cadherin* (*Ecad*). The results showed that the mRNA expression of *Ecad* increased in the duodenum, yet decreased in the colon after PS50 and PS500 exposure (Fig. 5I). The *Ecad* expression remained unchanged in the jejunum and ileum after PS50 and PS500 exposure (Fig. 5I). PS500 and the mixture of PS50 and PS500, but not PS50, increased the most in the TJP mRNA expression in the duodenum and colon (Fig. 5K, N). However, PS50 and PS500 did not change the TJP mRNA expression in the jejunum and ileum (Fig. 5L, M). Of note, most of the TJP mRNA expression increased in the jejunum when PS50 and PS500 were presented as a mixture, compared with the control group (Fig. 5L). In addition, analyses showed an association between the TJPs’ and mucins’ mRNA expressions in the duodenum, jejunum and colon (Fig. 5O, P, R), but not in the ileum (Fig. 5Q).

Creatinine, 4 kDa dextran, and 70 kDa dextran were used to probe pore, leak, and unrestricted permeability pathways. Intestinal permeability to creatinine and 70 kDa dextran was elevated after exposure to either PS50, PS500 or a mixture of PS50 and PS500 (Fig. 5J). The intestinal permeability to 4 kDa dextran was elevated after exposure to either PS500 or a mixture of PS50 and PS500 (Fig. 5J). The enhanced 70 kDa dextran permeability suggested increased unrestricted pathway flux and epithelial damage. The enhanced creatinine and 4 kDa dextran fluxes were due to the increase in unrestricted pathway permeability, as the creatinine or 4 kDa dextran fluxes returned to baseline when normalized to the 70 kDa dextran flux (Fig. 5J). We used five indexes to evaluate gut barrier conditions: pathological change (PC), mucin secretion (MU), reaction oxygen species (ROS), apoptosis (AP), and tight junction (TJ). We used Sankey diagrams to summarize the above results and found that most of the gut barrier damage had been caused by increased ROS and apoptosis in the four mouse intestinal segments (Fig. 5S-V).

### ROS neutralization reduced epithelial cell apoptosis and PS biodistribution

**Fig. 6:**
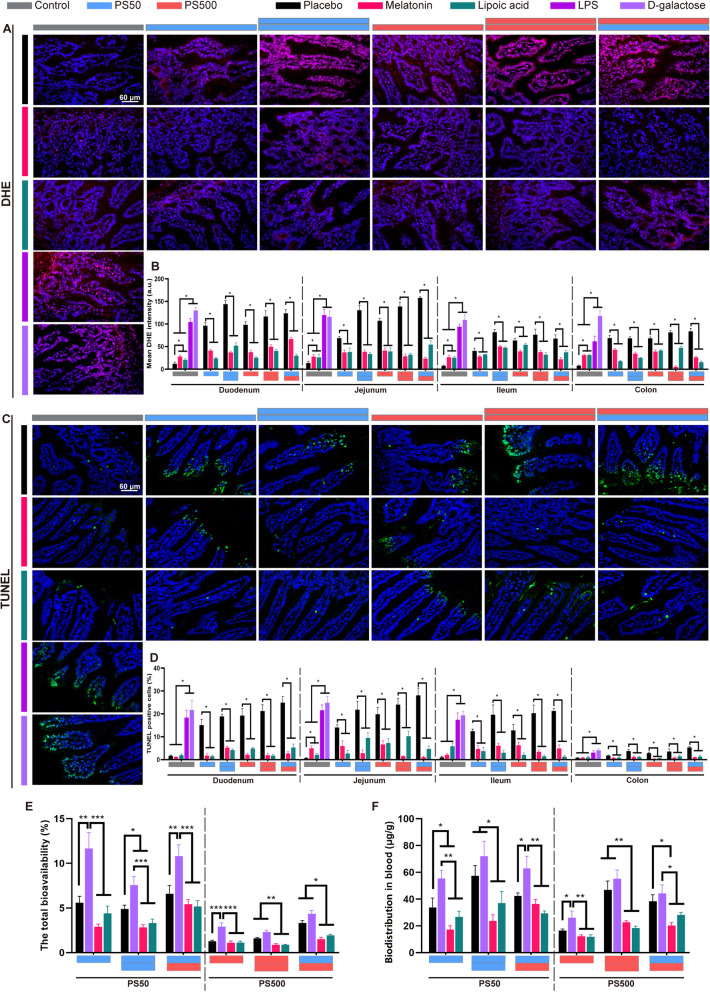
ROS neutralization reduced epithelial cell apoptosis and PS particles biodistribution in the intestine. **A** Representative merged images of the jejunum sections stained with DHE to assess the ROS generation after oxidant or antioxidant treatment. The ROS was stained in red and the nucleus was stained with DAPI in blue. **B** The quantification of mean DHE intensity in the four intestinal segments was shown. **C** Representative merged images of the jejunum sections stained with TUNEL to assess the cell apoptosis after oxidant or antioxidant treatment. The apoptosis cells were stained in green and the nucleus was stained with DAPI in blue. **D** The quantification of TUNEL positive cell rate in the four intestinal segments. **E** Total bioavailability of PS50 and PS500 after oxidant or antioxidant treatment. **F** Total accumulation of PS50 and PS500 in the intestine after oxidant or antioxidant treatment. *P < 0.05, **P < 0.01, and ***P < 0.001. Results were shown as means ± SE. Comparisons were made with ANOVA, followed by Tukey’s method (*n* = 5 per group). The color bars were used for grouping. The blue and red bar represented a dose of 250 mg/kg body weight for PS50 and PS500, respectively, two bars with same color represented a dose of 500 mg/kg body weight, and two bars with different colors represented the mix exposure group with a dose of 250 mg/kg body weight of each particle

Our results revealed a correlation between PS micro- and nanoplastics exposure, ROS generation, epithelial cell apoptosis and increased intestinal permeability. To elucidate their causalities, we explored whether inducing ROS generation could increase intestine epithelial cell apoptosis and permeability. The mice were pretreated by two oxidant agonists (lipopolysaccharide and D-galactose) 2 h before the PS micro- and nanoplastics exposure. Both of these oxidant agonists were effective in inducing ROS bursts and epithelial cell apoptosis in all four intestinal segments, compared to the placebo group (Figs. 6A, C and S10). Therefore, the intestinal permeability increased, as evidenced by an increasing PS50 and PS500 total bioavailability and blood biodistribution (Fig. 6E, F). Further, we explored whether removing ROS could reduce the PS-induced intestine epithelial cell apoptosis, and thereby restore the intestinal barrier’s permeability. The mice were pretreated with two antioxidants (melatonin and lipoic acid) 2 h before the PS micro- and nanoplastics exposure. Both of these antioxidants were effective in scavenging ROS in all four of the intestinal segments, compared to the placebo group (Figs. 6A, B and S10). Moreover, the intestine epithelial cell apoptosis decreased (Figs. 6C, D and S10). As a result, the intestinal permeability was restored, as evidenced by non-detectable increases in PS50 and PS500 total bioavailability and blood biodistribution, compared to the placebo group and oxidant agonist groups (Fig. 6E, F). The detailed PS50 and PS500 biodistribution in the brain, heart, lungs, liver, spleen, kidneys, testis and epididymis, stomach, and small and large intestines after oxidant and antioxidant treatment is shown in Figure S11.

### 28-day repeated dose oral exposure to PS micro- and nanoplastics has combined effects in mice

**Fig. 7:**
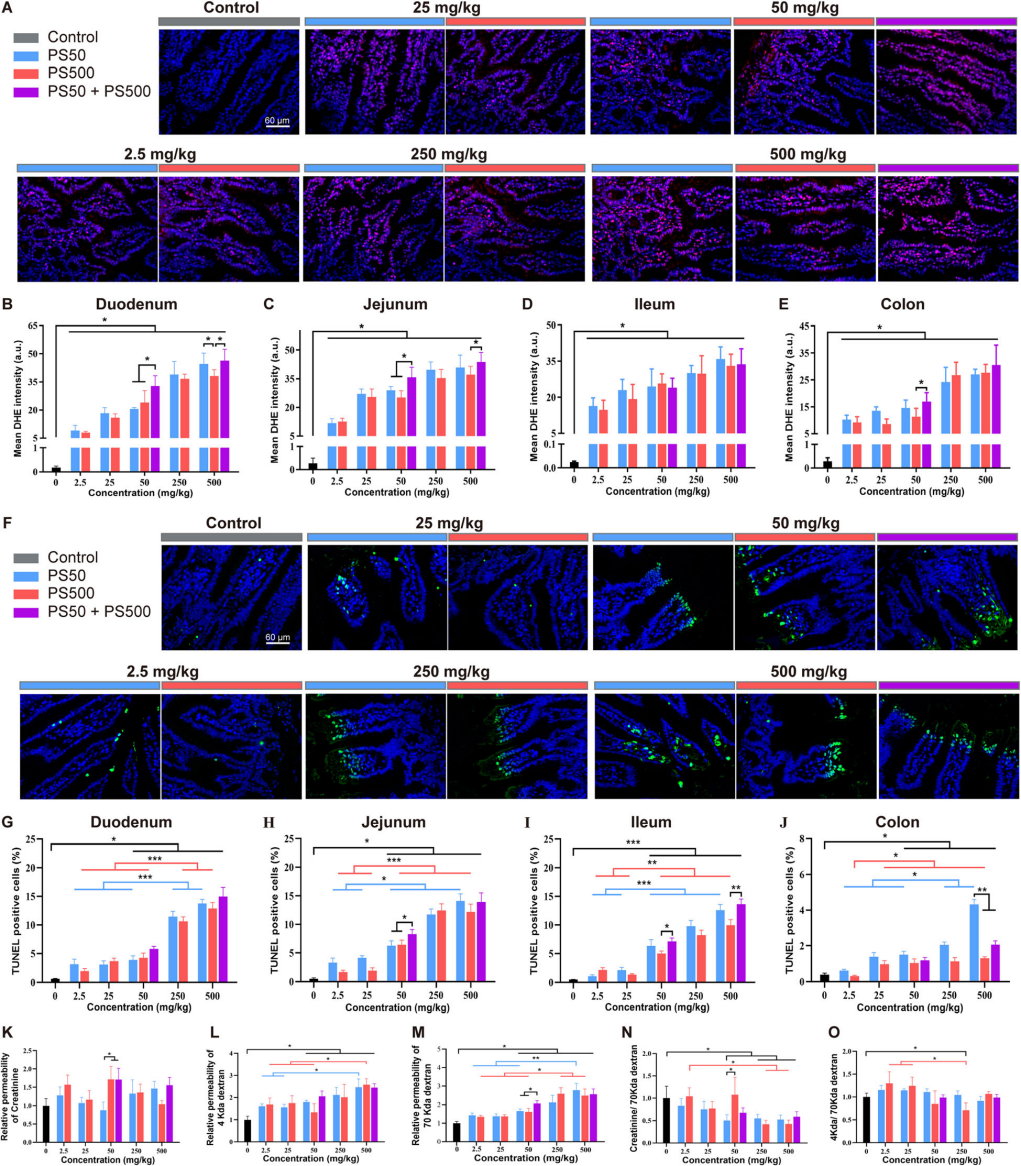
28-day repeated dose oral exposure to PS micro- and nanoplastics confirmed the combined effects. **A** Representative merged images of the jejunum sections stained with DHE to assess the ROS generation after repeated dose of PS particles exposure for 28 days. The ROS was stained in red and the nucleus was stained with DAPI in blue. The quantification of mean DHE intensity in **B**) duodenum, **C**) jejunum, **D**) ileum and **E**) colon was shown. **F** Representative merged images of the jejunum sections stained with TUNEL to assess the cell apoptosis after repeated dose of PS particles exposure for 28 days. The apoptotic cells were stained in green and the nucleus was stained with DAPI in blue. The quantification of TUNEL positive cell rate in **G**) duodenum, **H**) jejunum, **I**) ileum and **J**) colon was shown. The fluxes of **K**) creatinine, **L**) 4 kDa dextran, **M**) 70 kDa dextran in the intestine were quantified. The fluxes of **N**) creatinine and **O**) 4 kDa dextran were normalized to 70 kDa dextran flux. *P < 0.05. Results were shown as means ± SE. Comparisons were made with ANOVA, followed by Tukey’s method (*n* = 5 per group). The color bars were used for grouping. The exposure dose was shown by the color bars

To confirm the combined effects of PS micro- and nanoplastics in the intestine, and to explore a lowest PS dose for observed adverse effects in a longer PS micro- and nanoplastics exposure period, a 28-day repeated dose oral toxicity study in mice was conducted using a range of doses. Both PS50 and PS500 induced ROS generation (Figs. 7A-E and S12A, C, E), epithelial cell apoptosis (Figs. 7F-J and S12B, D, F) and increased intestinal permeability (Fig. 7K-O) in the four intestinal segments. We found the combined effects of PS micro- and nanoplastics in the 50 mg/kg body weight and 500 mg/kg body weight concentrations in the duodenum and jejunum. This was characterized as the mixture of PS50 and PS500 causing more ROS than that would have been caused by PS50 or PS500 alone (Fig. 7B-E). Of note, significant ROS generation was found at concentrations as low as 2.5 mg/kg body weight in the four intestinal segments (Fig. 7B-E). Increased epithelial cell apoptosis was found at the concentration of 50 mg/kg body weight in the 4 intestinal segments (Fig. 7G-J). However, we found no significant pathological changes in the four mouse intestinal segments (Figure S13). Parallel to the epithelial cell apoptosis, intestinal permeability to creatinine was elevated after exposure to PS500 or mixing PS50 and PS500 at a concentration of 50 mg/kg body weight (Fig. 7K). The intestinal permeability to 4 kDa dextran and 70 kDa dextran was elevated after exposure to PS50, PS500 or the mixture of PS50 and PS500 at the concentrations over 50 mg/kg body weight (Fig. 7L, M). The permeability to 70 kDa dextran increased after exposure to the mixture of PS50 and PS500 at the concentration of 50 mg/kg body weight, compared to exposure to PS50 or PS500 alone (Fig. 7M). The increased creatinine and 4 kDa dextran flux were due to the enhanced unrestricted pathway permeability, as neither the creatinine nor the 4 kDa dextran flux exceeded that of the control group, when normalized to 70 kDa dextran flux (Fig. 7N, O).

## Discussion

**Fig. 8:**
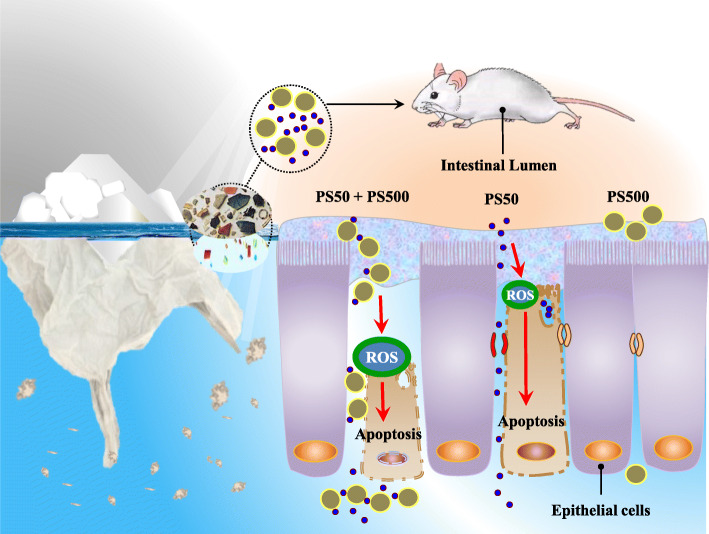
Schematic diagram of the present study. When microplastics are in the environment, more undetected nanoplastics are around. The current “microplastic exposure” is in fact the mixture of micro- and nanoplastic exposures. The PS micro- and nanoplastics affect the intestinal permeability in a size-dependent manner and change their biodistribution. More importantly, we found a combined toxicity of PS micro- and nanoplastics on the intestine. This manifested as the mixture of PS micro- and nanoplastics causing more severe intestinal barrier dysfunction, compared with the micro- or nanoplastics alone. Considering that most existing studies on PS micro- and nanoplastics have been conducted using a single particle size, the health risk of exposure to PS micro- and nanoplastics on organisms could be underestimated and in need of reevaluation accounting for the biological interaction between varying sizes of PS micro- and nanoplastics

The present study has investigated the interplay between PS micro- and nanoplastics that may affect their biodistribution and toxicities in mouse models. Under single or co-exposure conditions, we provided an overall perspective on the biodistribution of three PS sizes in mice. The PS sizes covered micro- to nano-scale. We found that an unexpected combined effect between PS50 and PS500 facilitates the biodistribution of both particles in mice. We confirmed that this was most likely due to the increased intestinal permeability caused by ROS-mediated epithelial cell apoptosis (Fig. 8). Given that most PS particles in environments appear as mixtures of sizes, and that the toxic effects related to PS particles have mostly been studied in single exposure conditions, the health risk of PS micro- and nanoplastics exposure in humans could be underestimated.

Many factors affect particle absorption, such as particle size [22, 35], material [36], surface charge [22] and hydrophilicity [17]. Particle size is one of the most effective factors affecting the biodistribution of the PS micro- and nanoplastics [22, 35]. We set the co-exposure dose in our biodistribution experiment at 500 mg/kg body weight, i.e., 250 mg/kg body weight for each two particle types. We selected such a high dose to increase the PS micro- and nanoplastic detectability in the organs, given the low expectations of bioavailability in the mice. The estimated oral bioavailability of PS50, PS500 and PS5000 that we report (i.e. 6.16%, 1.53 and 0.46%, respectively) suggested that the smaller the particles, the greater the proportion of PS particles that enter the body. This indicates a ubiquitous size dependency in regard to PS bioavailability in organisms, as similar effects have also been reported in zebrafish [20], early juvenile fish [21], in vitro intestinal cell models [22], and rats [23]. However, conflicting findings have also been reported. The highest uptake into Caco-2 cells has been found in 4 μm microplastics, comparing to 1 μm and 10 μm microplastics alone [18]. This indicates that the relationship between micro- and nanoplastics’ particle uptake and particle size remains in question and warrants further investigation.

En route to absorption through the intestine, the PS particles encounter a series of barriers, including the mucus layer, the tight junctions blocking paracellular passage, and the epithelial cells of the intestine [37]. The gel-like mucus layer is the first physical barrier to absorption in the intestine [38]. It has been reported that PS5000 decreases mucus secretion in the colon [13]. Similar to this finding, our results showed that exposure to PS50 and PS500 decreases mucus secretion in the colon. However, we found that PS50 exposure stimulates mucus secretion in the duodenum, jejunum and ileum. This stimulatory effect has also been found with other nanoparticles, e.g., silver nanoparticles, where it increases the amount of intestinal mucin by stimulating goblet cells [39]. There is a positive correlation between mucus secretion and mucin gene expression [40]. The opposite effects of the small and large intestines may due to the difference in mucin gene expression and responses to the PS particles. Because the small intestine performs more important functions in absorption, it could also have been that exposure to PS50 and PS500 caused protective compensatory responses in the intestinal mucus layer under our experimental conditions.

The paracellular flux is restricted by the tight junctions which are sealed between adjacent epithelial cells [41]. There are two pathways through an intact epithelial monolayer, termed the “pore” and “leak” pathways, which are regulated by TJPs [41]. The intestinal permeability increases when the tight junctions break [42]. However, our findings revealed that the TJP gene expression did not decrease; some even increased after PS micro- and nanoplastics exposure. Increased TJP mRNA expression is commonly found to act as a protective mechanism when the intestine faces xenobiotics [43, 44]. The increased *CLDN1* and *CLDN4* expression tightened the junctions after narenginin exposure in Caco-2 cells [43]. Increased intestinal epithelial *CLDN2* expression facilitates pathogen clearance [44]. More importantly, the micro- and nanoplastics could not cross the intestinal barrier efficiently via the above-mentioned two pathways since the diameters were as wide as ~ 0.6 nm and ~ 6 nm, respectively [45]. When the tight junctions or epithelial cells were damaged, a high capacity and permissive pathway termed “unrestricted” pathway formed [41, 44]. This allowed large subjects like large protein and bacteria to shoot across the intestine [41]. As expected, we found increased 70 kDa dextran flux and epithelial cell apoptosis in the small intestine in a dose-dependent manner after PS50 and PS500 exposure. PS50 induced more severe apoptosis in the epithelial cells than did PS500. Additionally, PS-induced cell dysfunctions were in line with other previous in vivo and in vitro studies in a size-dependent manner [10, 46]. Parallel to the epithelial cell apoptosis, we found increased ROS generation in the small intestine. ROS generation is a common cause of nanoparticle-induced cell apoptosis [47]. ROS generation caused by PS micro- and nanoplastics has been reported in multiple organisms, such as zebrafish [10], rats [48] and mice [49], and ROS clearance alleviates the mucosal injury and damage in the intestine [50]. Via two proven effective antioxidants—lipoic acid and melatonin [51], the causality between PS micro- and nanoplastics exposure, ROS generation, epithelial cell apoptosis, and increased intestinal permeability has been confirmed in this study by clearing ROS during PS micro- and nanoplastic exposure. By using PS as one of the most abundant and typical micro- and nanoplastics [52, 53], our results draw an overall perspective that micro- and nanoplastics may induce oxidative stress in the small intestine, resulting in epithelial cell apoptosis and loss of the intestinal barrier. This led to increased intestinal permeability and micro- and nanoplastics absorption.

The absorption of both PS50 and PS500 was enhanced in the mice when PS50 was mixed with PS500, indicating that PS50 and PS500 have combined effects. This is possibly due to enhancing toxicity on the small intestine by inducing more ROS generation than PS50 or PS500 exposure alone when these two PS particles were mixed. The underlying mechanisms of the combined effects are unclear. However, an in vitro study has reported that the bigger nanoparticles promote the cellular uptake of the smaller nanoparticles with a total uptake increase of particles in the cells [32]. Additionally, some physicochemical properties could have changed when micro-particles mixed with nanoparticles [29, 34, 54, 55], which may also have affected their toxicities. The increased uptake of the “more-toxic” PS50 and the total particle count may be the cause of the enhanced toxicity in the mixture of PS50 and PS500.

The estimated daily intake (EDI) of microplastics with diameters between 0.5–10 μm for adults is 40.1 μg/kg body weight/day [56]. A much higher estimate proposes 5 g microplastics with diameters below 1 mm per week for human ingestion [57]. That is approximately 10 mg/kg body weight/day by taking 70 kg as the body weight of an adult into account. Furthermore, it is likely that the particle counts will be much higher if we account for the nanoplastics [28]. These nanoplastics have often been ignored due to the limitations of detection methods [58]. We conducted a 28-day repeated dose oral toxicity study on PS micro- and nanoplastics with a range of concentrations from 2.5 mg/kg body weight to 500 mg/kg body weight. We set the high dose of 500 mg/kg body weight to identify hazards and the low dose of 2.5 mg/kg body weight to approach the environmental level. Significant ROS generation was found in PS concentrations as low as 2.5 mg/kg body weight. Increased epithelial cell apoptosis was found in concentrations of PS over 50 mg/kg body weight. This indicated that PS micro- and nanoplastics can generate ROS in the intestine at a low concentration, but epithelial cell apoptosis occurs only when a considerable amount of ROS is produced after high PS micro- and nanoplastics exposure. Considering the combined effects of PS micro- and nanoplastics on the intestine, and that most human PS micro- and nanoplastics intake appears as a mixture of multiple sizes of PS particles [5, 6], the health risk of PS particles exposure in humans should not be underestimated.

## Conclusions

PS micro- and nanoplastics exposure causes intestinal barrier dysfunction by ROS-mediated epithelial cell apoptosis in a size-dependent manner. This affects the absorption and biodistribution of PS micro- and nanoplastics in body organs. More importantly, we found a combined toxicity of PS micro- and nanoplastics on the intestine. This manifested as the mixture of PS micro- and nanoplastics causing more severe intestinal barrier dysfunction, which therefore changed their biodistribution, compared with PS micro- or nanoplastics alone. Considering that most existing studies on PS micro- and nanoplastics have only examined a single particle size, the health risks associated with exposure to PS micro- and nanoplastics in organisms could be underestimated.

## Materials and methods

### Chemicals and microplastics

Chemicals were purchased from Sigma-Aldrich (Taufkirchen, Germany), Merck (Darmstadt, Germany), or Carl Roth (Karlsruhe, Germany), unless otherwise indicated. The unmodified PS particles, including NF, RF and GF particles were purchased from Magsphere (Pasadena, California, USA). All of the PS particle densities were 1.05 g/mL provided by the Certificate of Analysis.

### Particle characterization

Particle sizes were morphologically characterized by SEM (Zeiss Supra55, Carl Zeiss AG, Germany), following previously described protocols [22]. The PS suspensions’ size distribution and zeta-potential were detected by a Dynamic Light Scattering with a Zetasizer Nano ZS (Malvern Panalytical GmbH, Kassel, Germany). Each PS particle type was suspended in distilled water, simulated gastric or intestinal fluids at a concentration of 0.25 mg/mL. We prepared the simulated gastric and intestinal fluids by following previously described protocols [59]. We determined each PS particle type’s in vitro fluorescence leakage by incubating 1 mL PS suspensions (0.25 mg/mL) in distilled water, and simulated gastric or intestinal fluids at 37 °C for 1, 6, 12 and 24 h by following previously described protocols [32].

### Mouse husbandry

A total of 455 C57BL/6 J mice (6-week-old, body weight 18–20 g, 400 males, 55 females, specific pathogen free) were used in this study. The mice were provided by the Guangdong Medical Laboratory Animal Center (Guangzhou, China). All animals were housed and acclimated to the new environment for 7 days in a temperature- (23–25 °C) and humidity- (50–60%) controlled room under a 12 h light/dark cycle (lights on from 8:00 a.m. to 8:00 p.m.). Food and sterilized water were provided ad libitum. The exposure conditions and number of mice for each experimental group are listed in Table 1. Mice were randomly divided into each group based on their body weights at the beginning of the experiment.

**Table 1:** The mice experimental design

Experiment	Group No.	PS50 (mg/kg body weight)	PS500 (mg/kg body weight)	PS5000 (mg/kg body weight)	Male (*n*)	Female (*n*)
Biodistribution experiment^a)^	1	0	0	0	10	5
	2	250 (RF)	0	0	10	5
	3	250 (RF)	125 (GF)	0	10	5
	4	250 (RF)	250 (GF)	0	10	5
	5	250 (RF)	500 (GF)	0	10	5
	6	0	250 (RF)	0	10	5
	7	125 (RF)	250 (GF)	0	10	5
	8	500 (RF)	250 (GF)	0	10	5
	9	0	0	250 (RF)	10	5
	10	250 (RF)	0	250 (GF)	10	5
	11	0	250 (RF)	250 (GF)	10	5
Toxicity effects on intestinal barrier experiment^b)^	1	0	0	0	10	0
	2	250 (NF)	0	0	10	0
	3	500 (NF)	0	0	10	0
	4	0	250 (NF)	0	10	0
	5	0	500 (NF)	0	10	0
	6	250 (NF)	250 (NF)	0	10	0
Oxidant and antioxidant treatment experiment^c)^	1	0	0	0	25	0
	2	250 (NF)	0	0	15	0
	3	500 (NF)	0	0	15	0
	4	0	250 (NF)	0	15	0
	5	0	500 (NF)	0	15	0
	6	250 (NF)	250 (NF)	0	15	0
28-day repeated dose oral toxicity study^d)^	1	0	0	0	10	0
	2	2.5 (NF)	0	0	10	0
	3	25 (NF)	0	0	10	0
	4	50 (NF)	0	0	10	0
	5	250 (NF)	0	0	10	0
	6	500 (NF)	0	0	10	0
	7	0	2.5 (NF)	0	10	0
	8	0	25 (NF)	0	10	0
	9	0	50 (NF)	0	10	0
	10	0	250 (NF)	0	10	0
	11	0	500 (NF)	0	10	0
	12	25 (NF)	25 (NF)	0	10	0
	13	250 (NF)	250 (NF)	0	10	0

^a)^ Results in Fig. 3 and 4 and Figures S2, S3, S4, S5, S6, S7, and S8; ^b)^ Results in Fig. 5 and Figure S9; ^c)^ Results in Fig. 6 and Figures S10 and S11; ^d)^ Results in Fig. 7 and Figures S12 and S13. NF, non-fluorescence; RF, red fluorescence; GF, green fluorescence

### Biodistribution experiment

We treated the mice with a single oral gavage with either double distilled water, or with PS micro- and nanoplastics in double distilled water, at a volume of 20 mL/kg body weight after fasting (from both solids and liquids) for 12 h. At the end of 24 h of exposure, we anesthetized the mice and collected the blood from the retroorbital plexus in an ethylene diamine tetraacetic acid anticoagulation tube. After perfusion with saline, we terminated the animals by cervical dislocation and collected tissue samples on ice. We cut the small intestines into the duodenum, jejunum and ileum, and the large intestine into the cecum and colon. In order to measure the residual of the PS micro- and nanoplastics in the intestine after exposure, we did not wash the contents of either the small or the large intestines. We froze the tissue samples immediately in liquid nitrogen, and then stored them at − 80 °C until use.

### 28-day repeated dose oral toxicity study in mice

After fasting (from both solids and liquids) for 12 h, we treated the mice with a single oral gavage, daily, with either double distilled water, or with PS micro- and nanoplastics in double distilled water at a volume of 20 mL/kg body weight, for 28 consecutive days. Before and during the exposure period (between 8:00 and 9:00 a.m. every 7 days), we weighed the mice. We adjusted the gavage volumes by the mice’s body weights every 7 days. At the end of exposure, we anesthetized the mice (0.3% pentobarbital sodium, intraperitoneal) and perfused them with saline. Then, we terminated the animals by cervical dislocation, and collected the tissue samples on ice. We cut the small intestines into the duodenum, jejunum and ileum, and the large intestines into the cecum and colon. Next, we washed the intestinal contents and cut each section of the intestine into halves. We immediately froze each intestine section in liquid nitrogen and then stored them at − 80 °C until use. We fixed the remaining half in 4% paraformaldehyde for pathological examination.

### Oxidant and antioxidant treatment

Individual oxidant and antioxidant compounds were dissolved in H_2_O or suspend in 1% sodium carboxymethylcellulose (CMC). Mice were treated by a single oral gavage with the oxidants or antioxidants 2 h before exposure to microplastics or nanoplastics. The oxidants or antioxidants were used at the following final doses: D-galactose, 1000 mg/kg body weight (dissolved in H_2_O); lipopolysaccharide, 10 mg/kg body weight (dissolved in H_2_O); lipoic acid, 100 mg/kg body weight (suspended in 1% CMC); melatonin, 40 mg/kg body weight (suspended in 1% CMC).

### RNA isolation and quantitative polymerase chain reaction analysis

Frozen animal tissues were homogenized in Trizol (Invitrogen, CA, USA), and total RNA was isolated following the manufacturer’s recommended protocol. For quantitative polymerase chain reaction (qPCR) analysis, the mRNA cDNA was synthesized using an Evo M-MLV One Step RT-PCR Kit (Accurate Biotechnology, China) following the manufacturer’s recommended protocols. The primers for mRNA (Table S2) were designed and synthesized by Tsingke Biological Technology (Beijing, China). qPCR was performed with SYBR® Green Premix Pro Taq HS qPCR Kit (Accurate Biotechnology) on the ABI QuantStudio™ 6 flex (Applied Biosystems, USA). The qPCR reaction conditions were as follows: 30 s at 95 °C, followed by 40 cycles of 5 s at 95 °C and 34 s at 60 °C. Primer specificity was confirmed by melting curve analysis, which showed a single product with the appropriate T_m_ for each primer set. All samples were analyzed in quadruplicate. The relative expression of *TJP* mRNA was normalized by *Ecad* and the others were normalized by *β-actin*. Gene relative expression was determined with the 2^-ΔΔCT^, compared with the control.

### Fluorescence imaging in vivo and ex vivo

At 1, 6, and 12 h post-gavage, mice were euthanized with isoflurane and underwent in vivo fluorescence imaging using an In-Vivo Multispectral (MS) FX PRO system (Bruker, Billerica, MA, USA) with excitation = 530 nm and emission = 600 nm for RF and excitation = 470 nm and emission = 535 nm for GF. At 24 h post-gavage, the mice were anesthetized and terminated. Organs and tissues were extracted and underwent ex vivo fluorescence imaging at the same conditions as above.

### Fluorescence detection in organ homogenate

Frozen animal tissues were weighted and homogenized in a digestive solution consisting of 1 g/L proteinase K (Python biotech, China), 5 g/L SDS, 23 g/L Na_2_HPO_4_, and 4.6 g/L NaH_2_PO_4_ using a FastPrep-24 Sample Preparation System (MP Biomedicals, Solon, OH, USA). We used 2.5 mL digestive solution per gram of tissue to digest the tissues. The homogenate was then digested in water bath at 37 °C for 12 h, resulting in a clear digested tissue solution. The digested solution of each tissue in the control group was mixed and diluted with double distilled water based on need. For intestines, we diluted the digested solution 30 times; for other organs, we diluted the digested solution 5 times. In addition, we diluted the blood with 12 mL double distilled water per gram of blood. Serial dilutions of each fluorescence particle in each tissue solution and in the blood were prepared and measured by a fluorescence spectrophotometer (Tecan Spark, Austria) with excitation = 538 nm and emission = 584 nm for RF and excitation = 480 nm and emission = 525 nm for GF. A standard curve for each fluorescence particle in each tissue and the blood was calculated in the corresponding organ homogenate of the control group animals. The digested solution of each tissue, and the blood in the exposure group, were diluted just like the control group, with double distilled water, and measured under the same conditions. After 24 h exposure, we evaluated the PS micro- and nanoplastic biodistribution by the concentrations of the PS particles in each organ or blood. This was calculated as (particle weight) / (organ weight). We evaluated the PS micro- and nanoplastic bioavailability by the fraction (%) of administered particles that reached the major organs, including the brain, heart, lungs, liver, spleen, kidneys, reproductive organs, muscles, blood, and bone, and excluding the gastrointestinal tract. It was calculated as (total amount in the major organs and blood) / (total amount of gavage). We evaluated the PS micro- and nanoplastic accumulation in the intestines after 24 h exposure by the fraction (%) of residue particles remaining in the gastrointestinal tract. It was calculated as (total amount in the gastrointestinal tract) / (total amount of gavage).

### Fluorescence particle detection on tissue slides

Frozen animal tissues were postfixed overnight and the fixative was replaced with a series of 10, 20 and 30% sucrose solution (Sigma Aldrich). Then, the tissues were embedded in optimal cutting temperature compound (Tissue-Tek®, Sakura Finetek Japan Co., Ltd., Tokyo, Japan). Coronal sections were used for fluorescence detection. Slides were scanned by Pannoramic MIDI (3D HISTECH, Budapest, Hungary) and images were reviewed with Pannoramic Viewer (3D HISTECH).

### ROS detection and TUNEL fluorescence assay on tissue slides

In situ ROS and apoptosis detection was performed using DHE assay (Sigma-Aldrich) and TUNEL fluorescence assay, respectively, following previously described protocols with slight modifications [51]. For DHE staining, the intestinal sections were incubated with 20 mM DHE on a shaker at room temperature for 30 min in the dark. Then the slides were washed three times in PBS for 5 min, using a shaker at room temperature. For TUNEL staining, a Click-iT® TUNEL Alexa Fluor® 488 Imaging Assay (Invitrogen) was used according to the manufacturer’s instructions. Slides were immediately imaged with identical settings on an OLYMPUS BX53 fluorescence microscope (OLYMPUS, Japan) or scanned by Pannoramic MIDI (3D HISTECH). The images were viewed with Pannoramic Viewer (3D HISTECH).

### Histopathological analysis

Intestinal segments, including the duodenum, jejunum, ileum and colon, were cut into small pieces and fixed immediately in 4% (wt/vol) paraformaldehyde solution. The fixed intestinal tissues were then dehydrated in a rising series of ethanol, cleared in xylene, and embedded in paraffin wax at 56 °C. Coronal sections were used for H&E staining, AB-PAS staining and TUNEL staining. Slides were scanned by Pannoramic MIDI (3D HISTECH) and images were reviewed with Pannoramic Viewer (3D HISTECH).

### Image quantification

Image quantification was performed using ImageJ 1.52v (National Institute of Health, Bethesda, MD, USA). We calculated the mucus coverage ratio in each group as (pixels in the mucus area) / (total pixels area of each gut section), as described previously [13]. We calculated the DHE intensity in each group by measuring the fluorescent signal’s pixel intensity in each gut section, as described previously [51]. To quantify the cell apoptosis rate, the TUNEL positive cells from each view were counted and normalized to a 4′,6-diamidino-2-phenylindole (DAPI) count. At least 10 views from each slide were randomly selected and measured for statistical analysis.

### Intestinal permeability assay

Mice were administered with 0.2 mL saline containing 12 mg fluorescein isothiocyanate-4 kDa dextran, 8 mg rhodamine B isothiocyanate-70 kDa dextran, and 20 mg creatinine (Sigma) 3 h prior to the end of 24 h and 28 days PS micro- and nanoplastics exposure. Serum was harvested after 24 h of PS micro- and nanoplastics exposure. Creatinine was measured using a kit from Nanjing Jiancheng Bioengineering Institute (Jiangsu, China). Recovery of creatinine and fluorescent probes was measured in a Synergy HT plate reader (BioTek, Winooski, VT, USA) using freshly prepared standards. Fluorescein and rhodamine B fluorescence were measured using excitation wavelengths of 495 nm and 555 nm, and emission wavelengths of 525 nm and 585 nm, respectively.

### Statistical analysis

Data are presented as mean ± standard error of the mean (SE) unless indicated otherwise. All statistical analyses were performed in SPS PARTICLES 22.0 (IBM, Armonk, NY, USA). Comparisons among multiple exposure groups, and the corresponding control in each exposure experiment, were performed using a one-way analysis of variance (ANOVA) followed by a Tukey’s method. A probability (*P*) value < 0.05 was considered statistically significant. Statistical graphs were generated in Graph Pad Prism 8.0 (GraphPad Software, Inc., San Diego, CA, USA). The mouse model diagrams illustrating the PS organ biodistribution in the mice were generated using R [60] and the “gganatogram” package [61]. The sankey diagrams were generated in OriginPro 2019 (OriginLab Corp., Northampton, MA, USA).

## Supplementary Information

**Additional file 1: Figure S1.** Fluorescence leakage after incubated in the gastric and intestine juice. **Figure S2.** The dynamic biodistribution after single exposure with PS50, PS500 and PS5000. **Figure S3.** The standard curves of fluorescence intensity of PS particles in each organ or blood. **Figure S4.** The organ biodistribution after single exposure with PS50, PS500 and PS5000. **Figure S5.** The dynamic biodistribution after co-exposure with each two of PS50, PS500 and PS5000. **Figure S6.** The organ biodistribution after co-exposure with each two of PS50, PS500 and PS5000. **Figure S7.** The organ biodistribution after co-exposure with different proportions of PS50 and PS500. **Figure S8.** Histopathology confirmed the biodistribution in the lung, spleen and testis of mice. **Figure S9.** H&E, AB-PAS, DHE, TUNEL staining in the duodenum, ileum and colon after 24 h exposure. **Figure S10.** ROS generation and apoptosis in the duodenum, ileum and colon after oxidant or antioxidant treatment. **Figure S11.** The organ biodistribution of PS micro- and nanoplastics after oxidant or antioxidant treatment. **Figure S12.** ROS generation and apoptosis in the duodenum, ileum and colon after exposure for 28 days. **Figure S13.** H&E staining of the intestinal segments after exposure for 28 days. **Table S1**. Physical characteristics of PS micro- and nanoplastics. **Table S2.** Primer sequences for qPCR. **Table S3.** Body weight of a 28-day repeated dose oral toxicity study of PS micro- and nanoplastics exposure.

**Additional file 2.** These values correspond to the heatmaps in Fig. 5.

## Data Availability

The datasets supporting the conclusions of this article are included within the article and its Additional files 1 and 2.

## Abbreviations

AB-PAS: Alcian blue-periodic acid schiff
AP: Apoptosis
Cldn: Claudin
CMC: Carboxymethylcellulose
DAPI: 4′,6-diamidino-2-phenylindole
DHE: Dihydroethidium
Ecad: E-cadherin
GF: Green fluorescence
hr.: Hour
H&E: Hematoxylin-eosin
MU: Mucin secretion
NF: Non-fluorescence
*P*: Probability
PC: Pathological change
PDI: Polymer dispersity index
PS: Polystyrene
PS50: 50 nm PS
PS500: 500 nm PS
PS5000: 5000 nm PS
qPCR: Quantitative polymerase chain reaction
RF: Red fluorescence
ROS: Reactive oxygen species
SEM: Scanning electron microscope
TJP: Tight junction protein
TUNEL: TdT-mediated dUTP Nick-End Labeling
ZO: Zonula occludens
