# Supplementary material for: Underestimated health risks: polystyrene micro- and nanoplastics jointly induce intestinal barrier dysfunction by ROS-mediated epithelial cell apoptosis
Source: Part Fibre Toxicol. 2021 Jun 7;18:20. doi: 10.1186/s12989-021-00414-1 (PMC8186235; doi:10.1186/s12989-021-00414-1)
Supplement: Supplementary file 1 — Additional file 1: Figure S1. Fluorescence leakage after incubated in the gastric and intestine juice. Figure S2. The dynamic biodistribution after single exposure with PS50, PS500 and PS5000. Figure S3. The standard curves of fluorescence intensity of PS particles in each organ or blood. Figure S4. The organ biodistribution after single exposure with PS50, PS500 and PS5000. Figure S5. The dynamic biodistribution after co-exposure with each two of PS50, PS500 and PS5000. Figure S6. The organ biodistribution after co-exposure with each two of PS50, PS500 and PS5000. Figure S7. The organ biodistribution after co-exposure with different proportions of PS50 and PS500. Figure S8. Histopathology confirmed the biodistribution in the lung, spleen and testis of mice. Figure S9. H&E, AB-PAS, DHE, TUNEL staining in the duodenum, ileum and colon after 24 h exposure. Figure S10. ROS generation and apoptosis in the duodenum, ileum and colon after oxidant or antioxidant treatment. Figure S11. The organ biodistribution of PS micro- and nanoplastics after oxidant or antioxidant treatment. Figure S12. ROS generation and apoptosis in the duodenum, ileum and colon after exposure for 28 days. Figure S13. H&E staining of the intestinal segments after exposure for 28 days. Table S1. Physical characteristics of PS micro- and nanoplastics. Table S2. Primer sequences for qPCR. Table S3. Body weight of a 28-day repeated dose oral toxicity study of PS micro- and nanoplastics exposure. [file 12989_2021_414_MOESM1_ESM.docx]

**Underestimated health risks: Polystyrene micro- and nanoplastics jointly induce intestinal barrier dysfunction by ROS-mediated epithelial cell apoptosis**

Boxuan Liang ^a,1^, Yizhou Zhong ^a,1^, Yuji Huang ^a,1^, Xi Lin ^a,1^, Jun Liu ^a^, Li Lin ^a^, Manjiang Hu ^a^, Junying Jiang ^b^, Mingzhu Dai ^c^, Bo Wang ^a^, Bingli Zhang ^a^, Hao Meng ^a^, Jesse Justin J Lelaka ^a^, Haixia Sui ^d^, Xingfen Yang ^e,*^, Zhenlie Huang ^a,*^

*^a^Guangdong Provincial Key Laboratory of Tropical Disease Research, School of Public Health, Southern Medical University, Guangzhou 510515, PR China;*

*^b^Faculty of Preventive Medicine, School of Public Health, Guangdong Pharmaceutical University, Guangzhou 510006, PR China;*

*^c^Hunter Biotechnology, Inc., Hangzhou 310051, PR China;*

*^d^Division III of risk assessment, China National Center for Food Safety Risk Assessment, Beijing 100022, PR China;*

*^e^Food Safety and Health Research Center, School of Public Health, Southern Medical University, Guangzhou 510515, PR China.*

^1^ These authors contributed equally to this work as co-first authors.

^*^**Corresponding authors:**

Zhenlie Huang. Department of Toxicology, School of Public Health, Southern Medical University, 1023-1063 Shatai Nan Road, Guangzhou 510515, China. Phone: 86-20-61648415; Fax: 86-20-61648324. Email address: [huangzhenlie@126.com](mailto:huangzhenlie@126.com).

Xingfen Yang. Food Safety and Health Research Center, School of Public Health, Southern Medical University, 1023-1063 Shatai Nan Road, Guangzhou 510515, China. Phone: 86-20-61648301; Fax: 86-20-61648324. Email address: [xfyang@vip.163.com](mailto:xfyang@vip.163.com).

**Additional file 1: Figures S1-S13 and Tables S1-S3.**

**
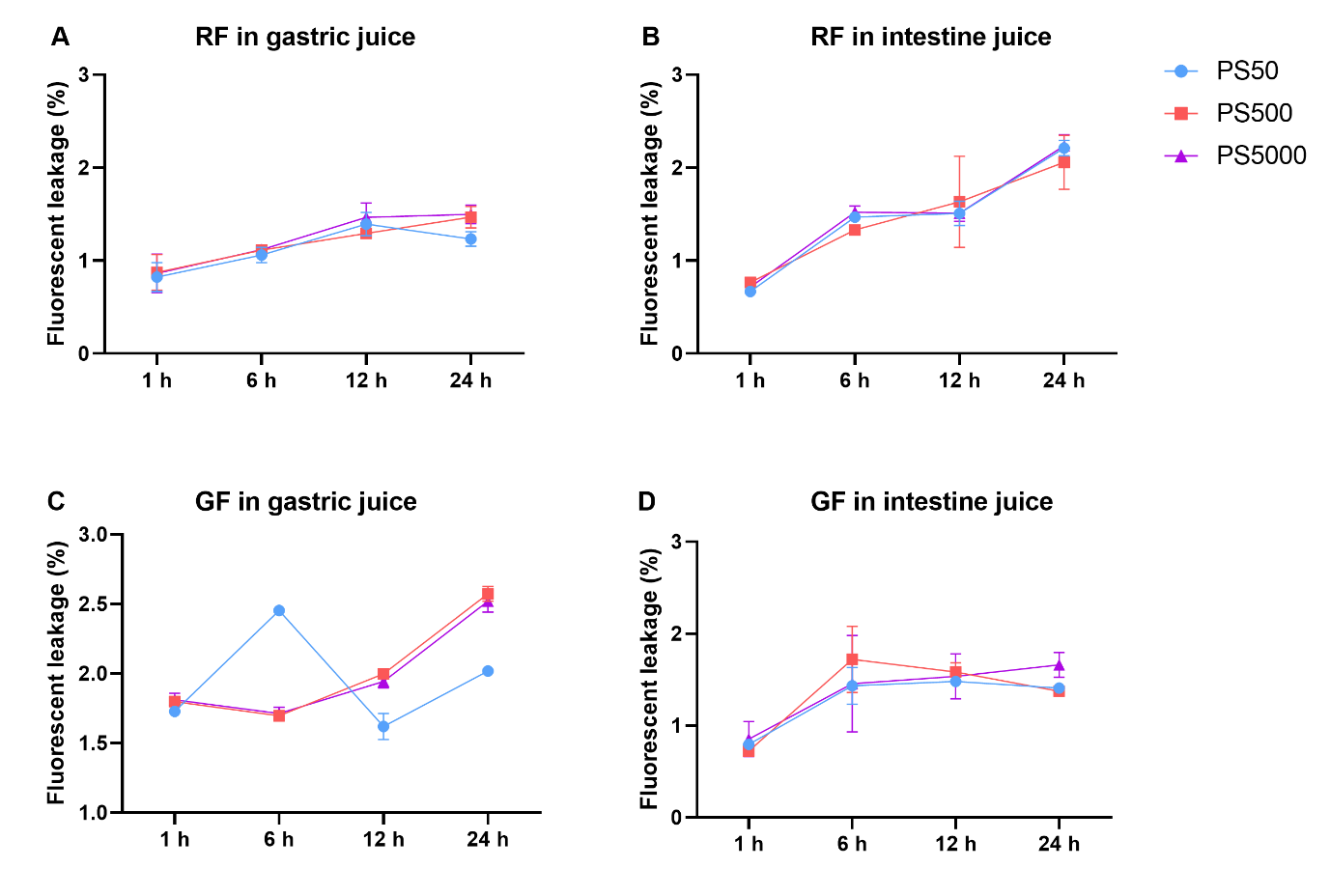
Figure S1.** Fluorescence leakage after incubated in the gastric and intestine juice. Fluorescence leakage from RF incubated in A) gastric juice and B) intestine juice. Fluorescence leakage from GF incubated in C) gastric juice and D) intestine juice. Values of fluorescence leakage are expressed as the means ± SE (*n* = 3 per group). RF, red fluorescence; GF, green fluorescence.

**
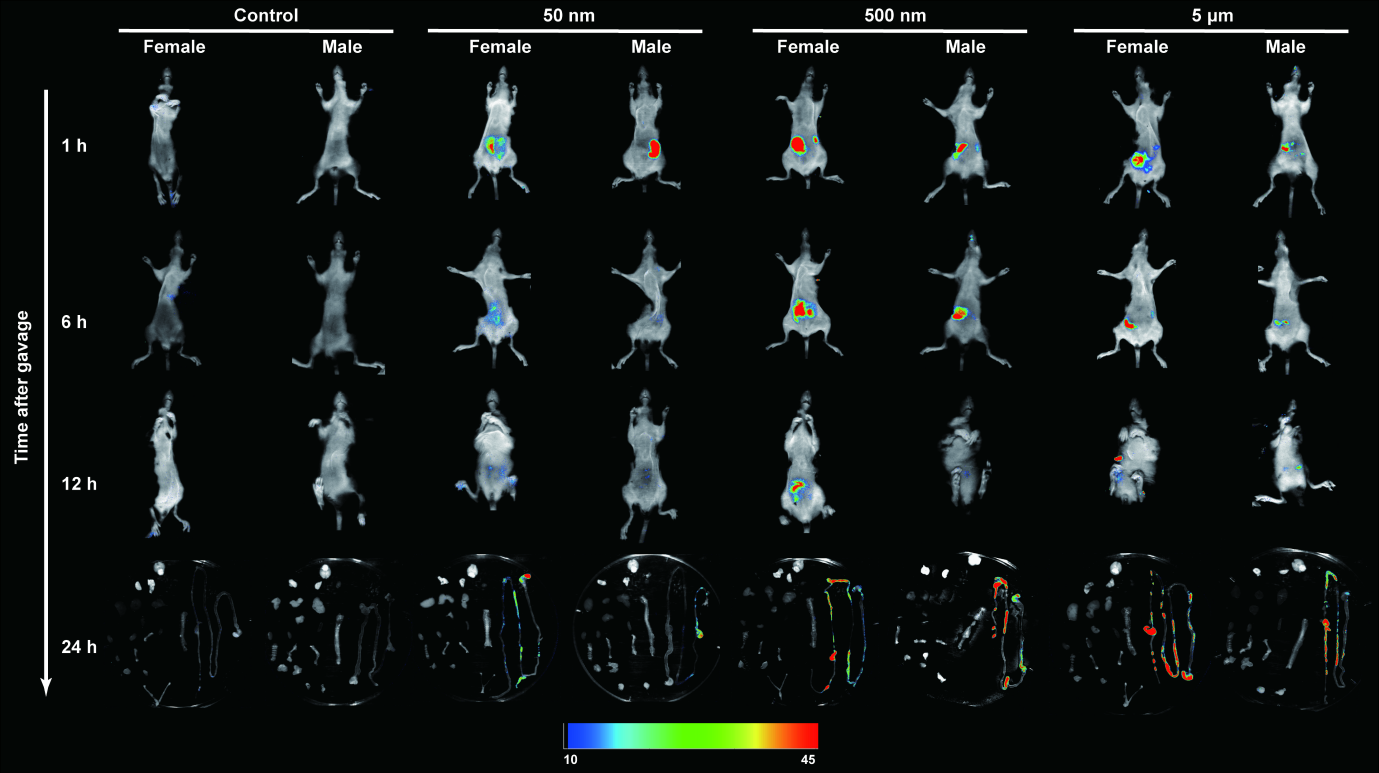
**

**Figure S2.** The dynamic biodistribution after single exposure with PS50, PS500 and PS5000. PS50, PS500 and PS5000 were detected by an *in-Vivo* fluorescence imaging by Multispectral (MS) FX PRO system. At 24 hr post-gavage, the mice were anesthetized and terminated. Organs and tissues were extracted and underwent *ex vivo* fluorescence imaging at the same conditions as the *in vivo* fluorescence imaging (*n* = 5 per group).

**
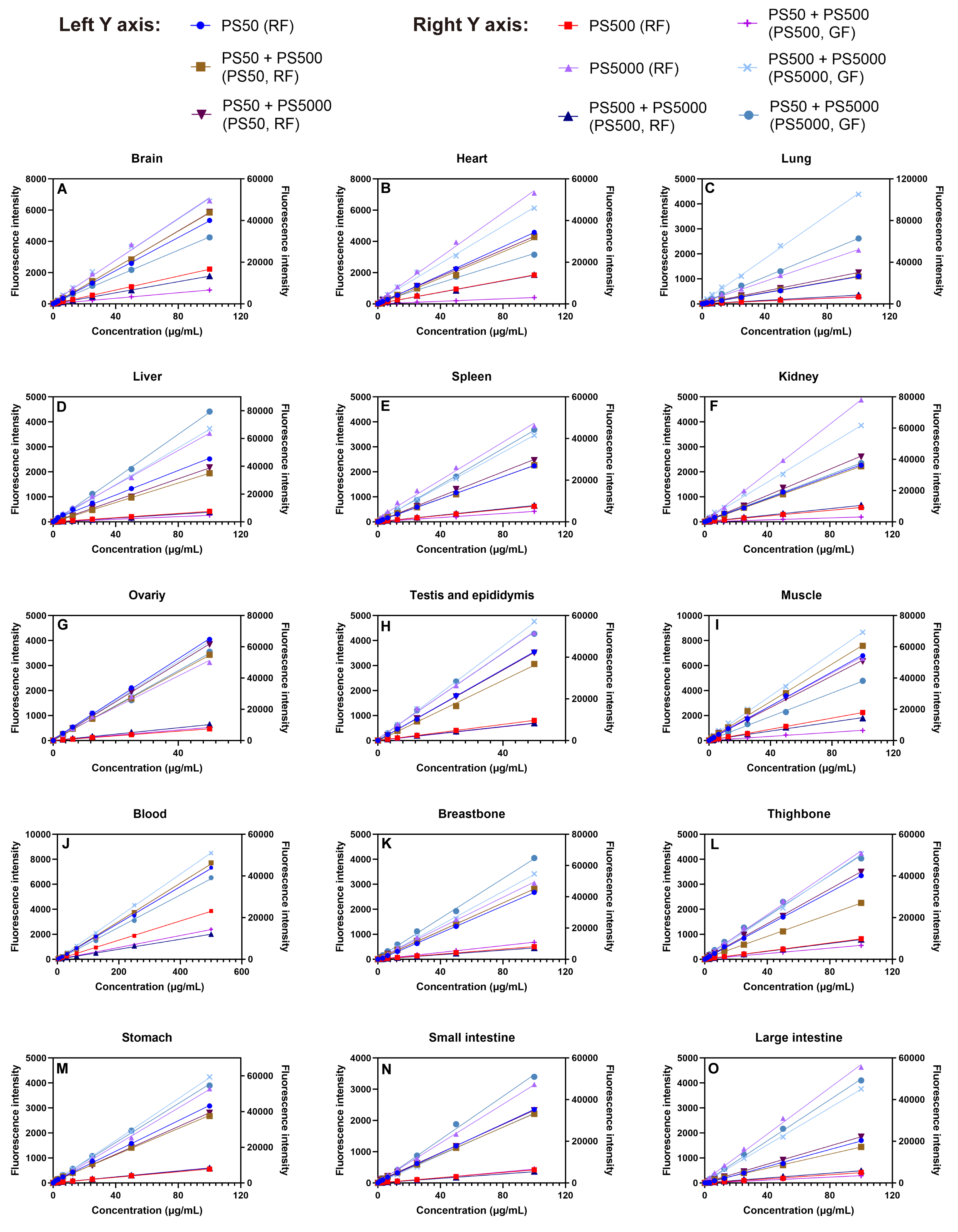
**

**Figure S3.** The standard curves of fluorescence intensity of PS particles in each organ or blood.

**
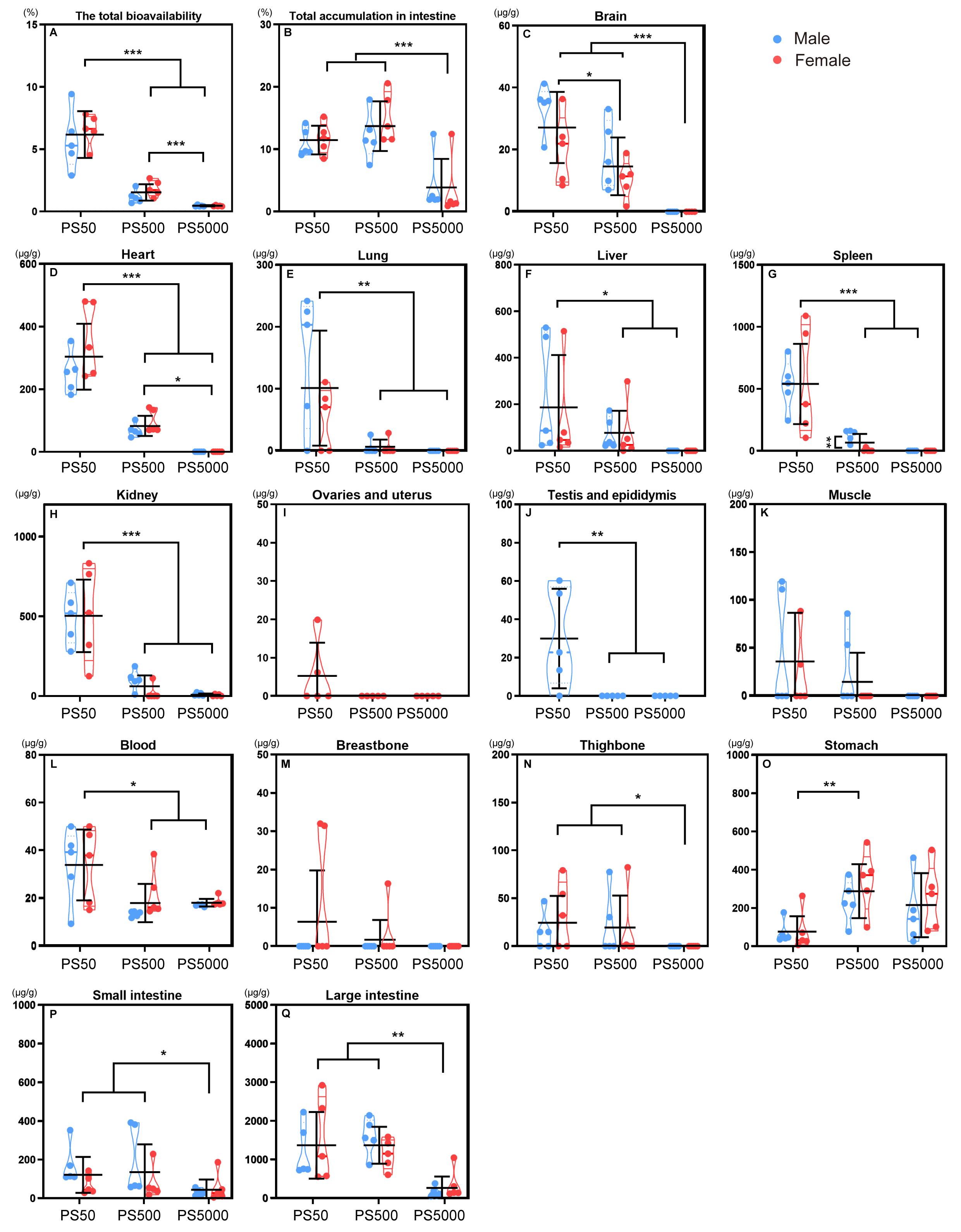
**

**Figure S4.** The organ biodistribution after single exposure with PS50, PS500 and PS5000. **P* < 0.05, ***P* < 0.01, and ****P* < 0.001. Results were showed as means ± SE. Comparisons were made with ANOVA, followed by a Tukey’s method (*n* = 5 per group).

**
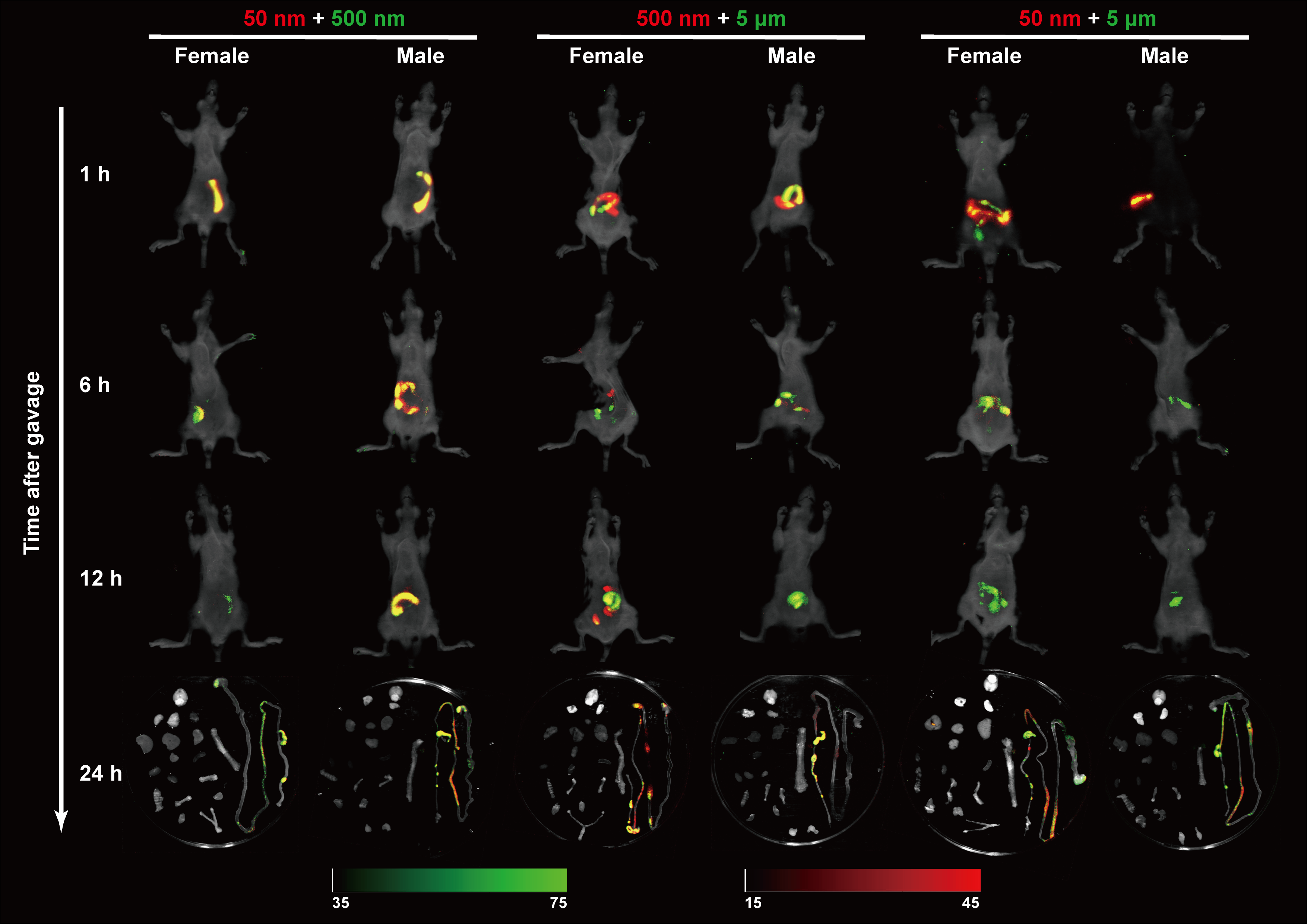
**

**Figure S5.** The dynamic biodistribution after co-exposure with each two of PS50, PS500 and PS5000. PS50, PS500 and PS5000 were detected by an *in-Vivo* fluorescence imaging by Multispectral (MS) FX PRO system. At 24 hr post-gavage, the mice were anesthetized and terminated. Organs and tissues were extracted and underwent *ex vivo* fluorescence imaging at the same conditions as the *in vivo* fluorescence imaging (*n* = 5 per group).

**
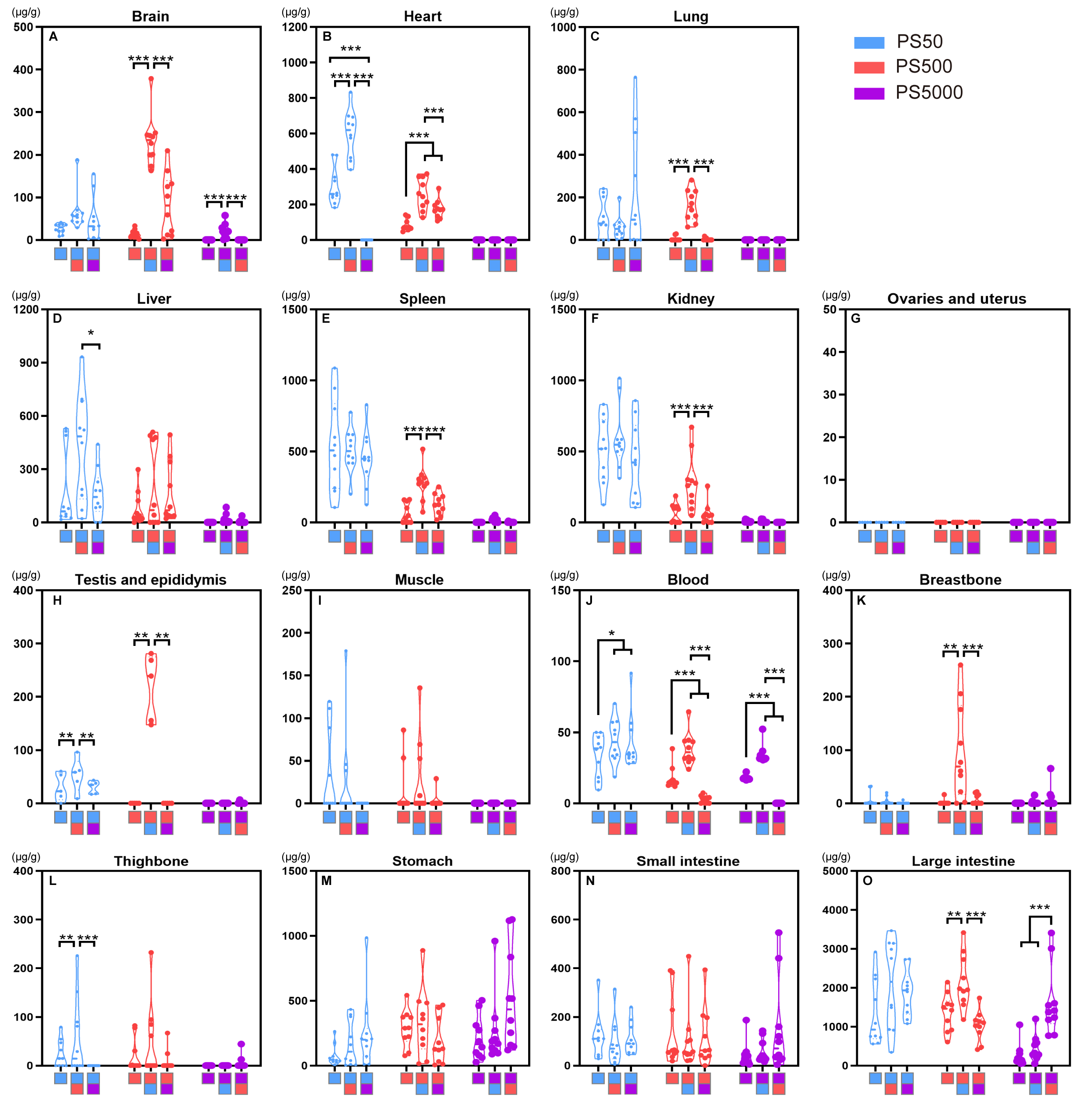
**

**Figure S6.** The organ biodistribution after co-exposure with each two of PS50, PS500 and PS5000. **P* < 0.05, ***P* < 0.01, and ****P* < 0.001. Results were showed as means ± SE. Comparisons were made with ANOVA, followed by a Tukey’s method (*n* = 5 per group). The color bars were used for grouping, one bar represented a dose of 250 mg/kg body weight, and two bars with different colors represented the mix exposure group with a dose of 250 mg/kg body weight of each particle.


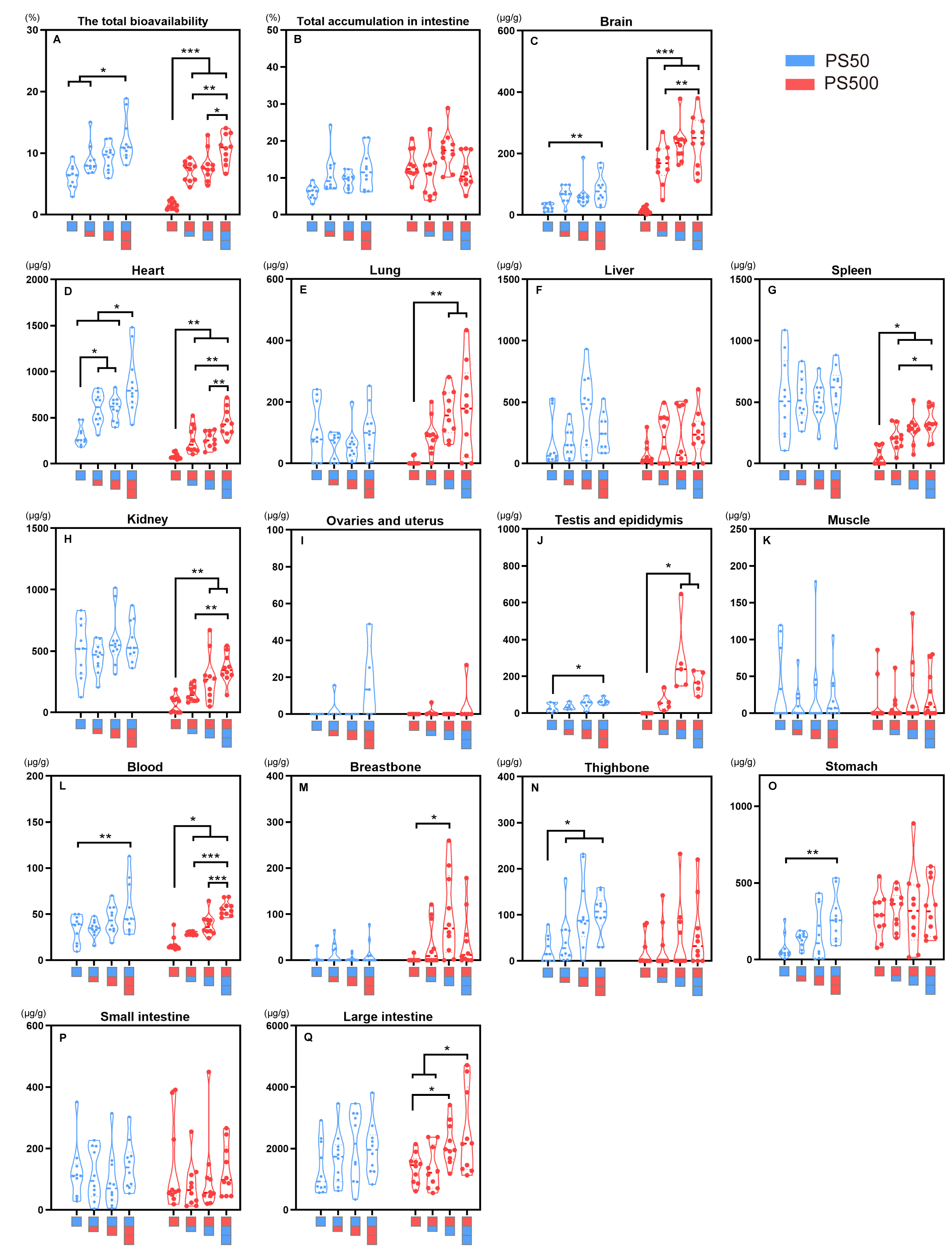


**Figure S7.** The organ biodistribution after co-exposure with different proportions of PS50 and PS500. **P* < 0.05, ***P* < 0.01, and ****P* < 0.001. Results were showed as means ± SE. Comparisons were made with ANOVA, followed by a Tukey’s method (*n* = 5 per group). The color bars were used for grouping, one bar represented a dose of 250 mg/kg body weight, and a half bar represented a dose of 125 mg/kg body weight. The combination of bars in each group represented the corresponding PS micro- and nanoplastics concentrations were used in the group.


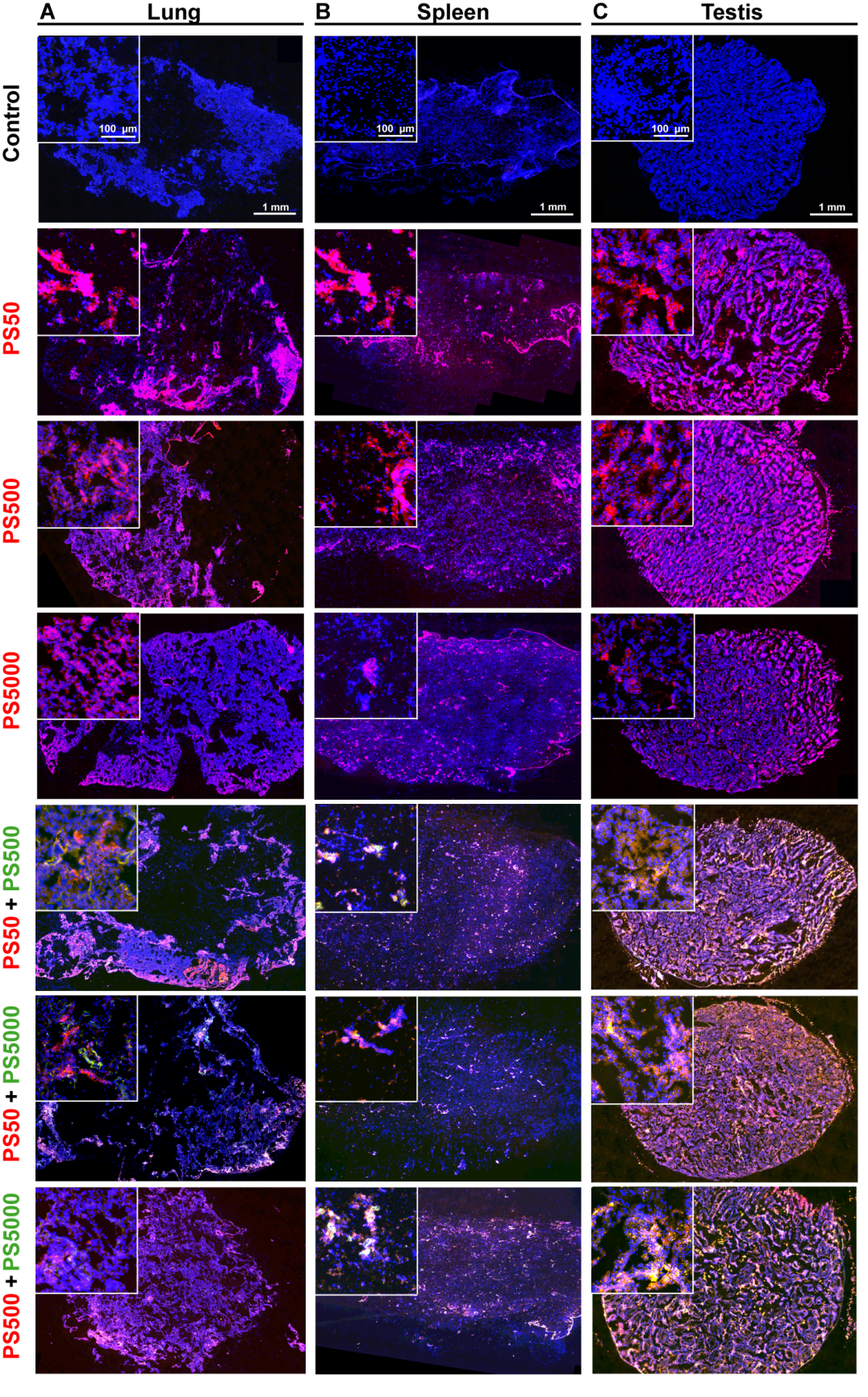


**Figure S8.** Histopathology confirmed the biodistribution in the lung, spleen and testis of mice. Representative merged fluorescence images of A) lung, B) spleen and C) testis (*n* = 5 per group). The font color represented the fluorescent color of the PS particle. The nucleus was stained with DAPI in blue.

**
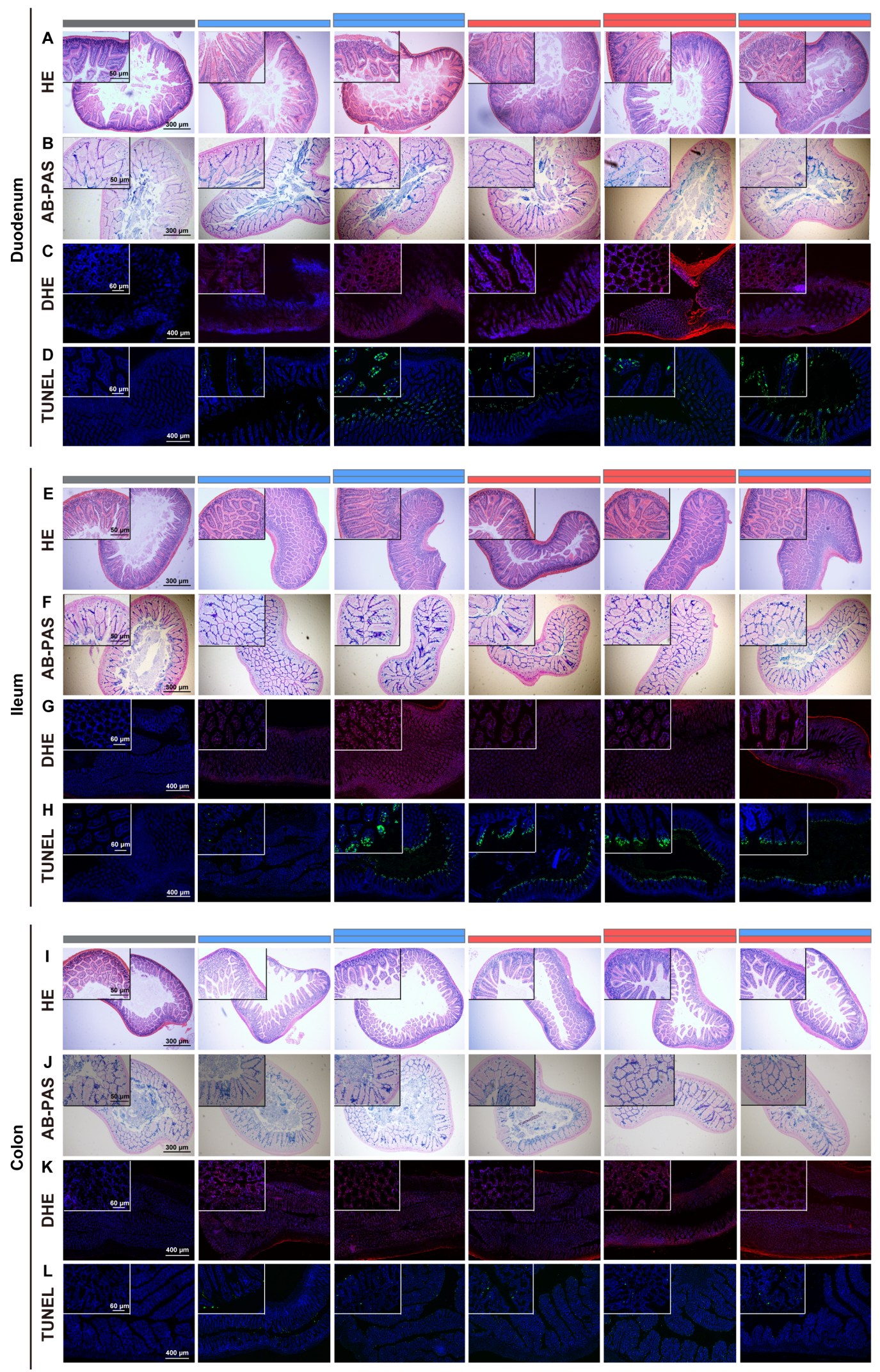
**

**Figure S9.** H&E, AB-PAS, DHE, TUNEL staining in the duodenum, ileum and colon after 24 hr exposure. Representative merged images of the duodenum sections A) stained with H&E to assess the morphological changes; B) stained with AB-PAS to assess the change of mucin secretion, the mucin was stained in blue; C) stained with DHE to assess the ROS generation, the ROS was stained in red and the nucleus was stained with DAPI in blue; D) stained with TUNEL to assess cell apoptosis, the apoptotic cells were stained in green and the nucleus was stained with DAPI in blue. Representative merged images of the ileum section E) stained with H&E; F) stained with AB-PAS, the mucin was stained in blue; G) stained with DHE, the ROS was stained in red and the nucleus was stained with DAPI in blue; H) stained with TUNEL, the apoptotic cells were stained in green and the nucleus was stained with DAPI in blue. Representative merged images of the colon section I) stained with H&E; J) stained with AB-PAS, the mucin was stained in blue; K) stained with DHE, the ROS was stained in red and the nucleus was stained with DAPI in blue; L) stained with TUNEL, the apoptotic cells were stained in green and the nucleus was stained in blue with DAPI. The color bars were used for grouping, one bar represented a dose of 250 mg/kg body weight, two bars with same color represented a dose of 500 mg/kg body weight, and two bars with different colors represented the mix exposure group with a dose of 250 mg/kg body weight of each particle. *n* = 5 per group.

**
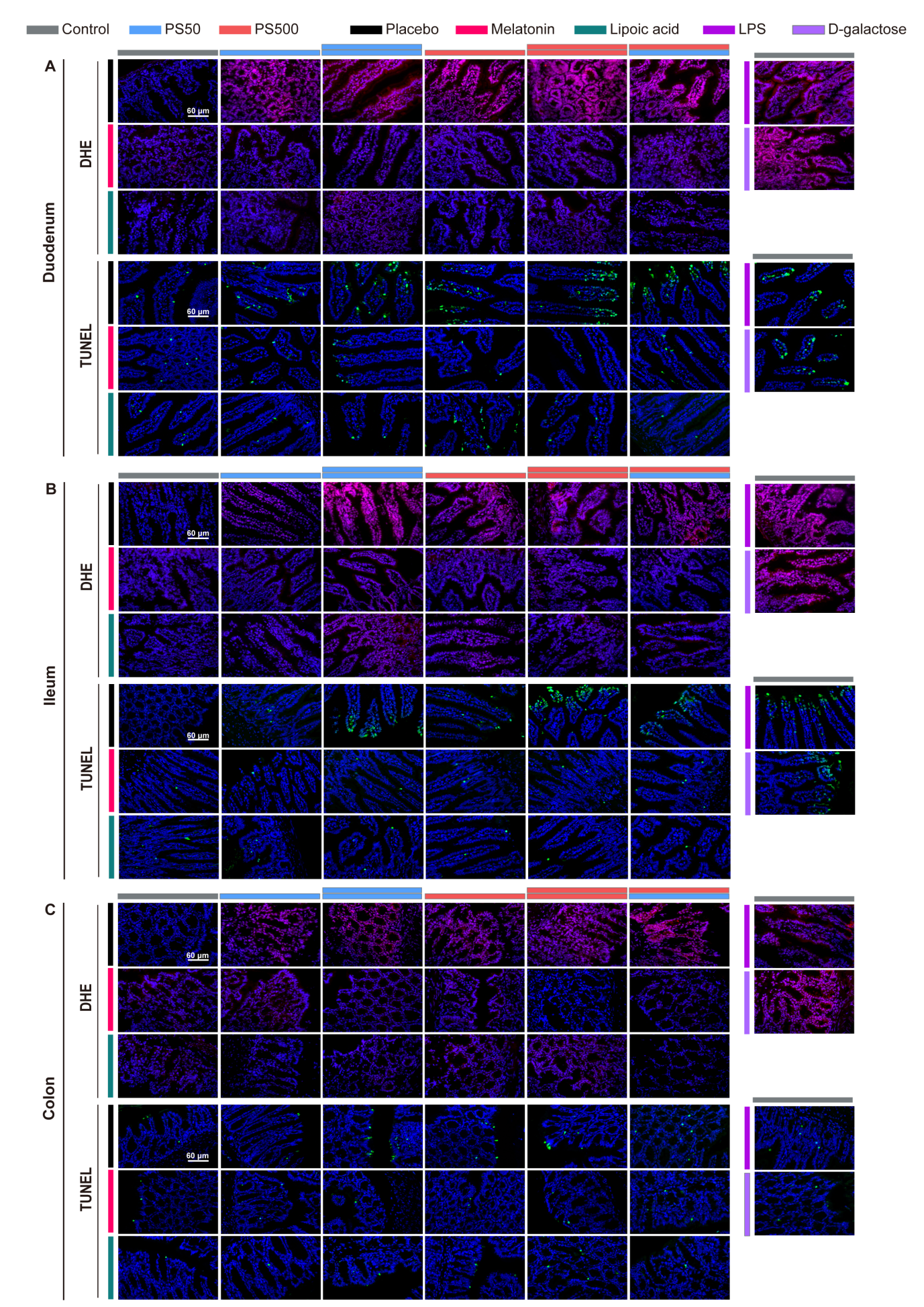
**

**Figure S10.** ROS generation and apoptosis in the duodenum, ileum and colon after oxidant or antioxidant treatment. A) Upper panel: Representative merged images of the duodenum sections stained with DHE to assess the ROS generation after oxidant or antioxidant treatment. The ROS was stained in red and the nucleus was stained with DAPI in blue. Lower panel: Representative merged images of the duodenum sections stained with TUNEL to assess the cell apoptosis after oxidant or antioxidant treatment. The apoptotic cells were stained in green and the nucleus was stained in blue with DAPI. B) Upper panel: Representative merged images of the ileum sections stained with DHE after oxidant or antioxidant treatment. The ROS was stained in red and the nucleus was stained with DAPI in blue. Lower panel: Representative merged images of the ileum sections stained with TUNEL after oxidant or antioxidant treatment. The apoptotic cells were stained in green and the nucleus was stained with DAPI in blue. C) Upper panel: Representative merged images of the colon sections stained with DHE after oxidant or antioxidant treatment. The ROS was stained in red and the nucleus was stained with DAPI in blue. Lower panel: Representative merged images of the colon sections stained with TUNEL after oxidant or antioxidant treatment. The apoptotic cells were stained in green and the nucleus was stained with DAPI in blue. The color bars were used for grouping, blue and red bars represented a dose of 250 mg/kg body weight for PS50 and PS500, respectively, two bars with same color represented a dose of 500 mg/kg body weight, two bars with different colors represented the mix exposure group with a dose of 250 mg/kg body weight of each particle. *n* = 5 per group.

**
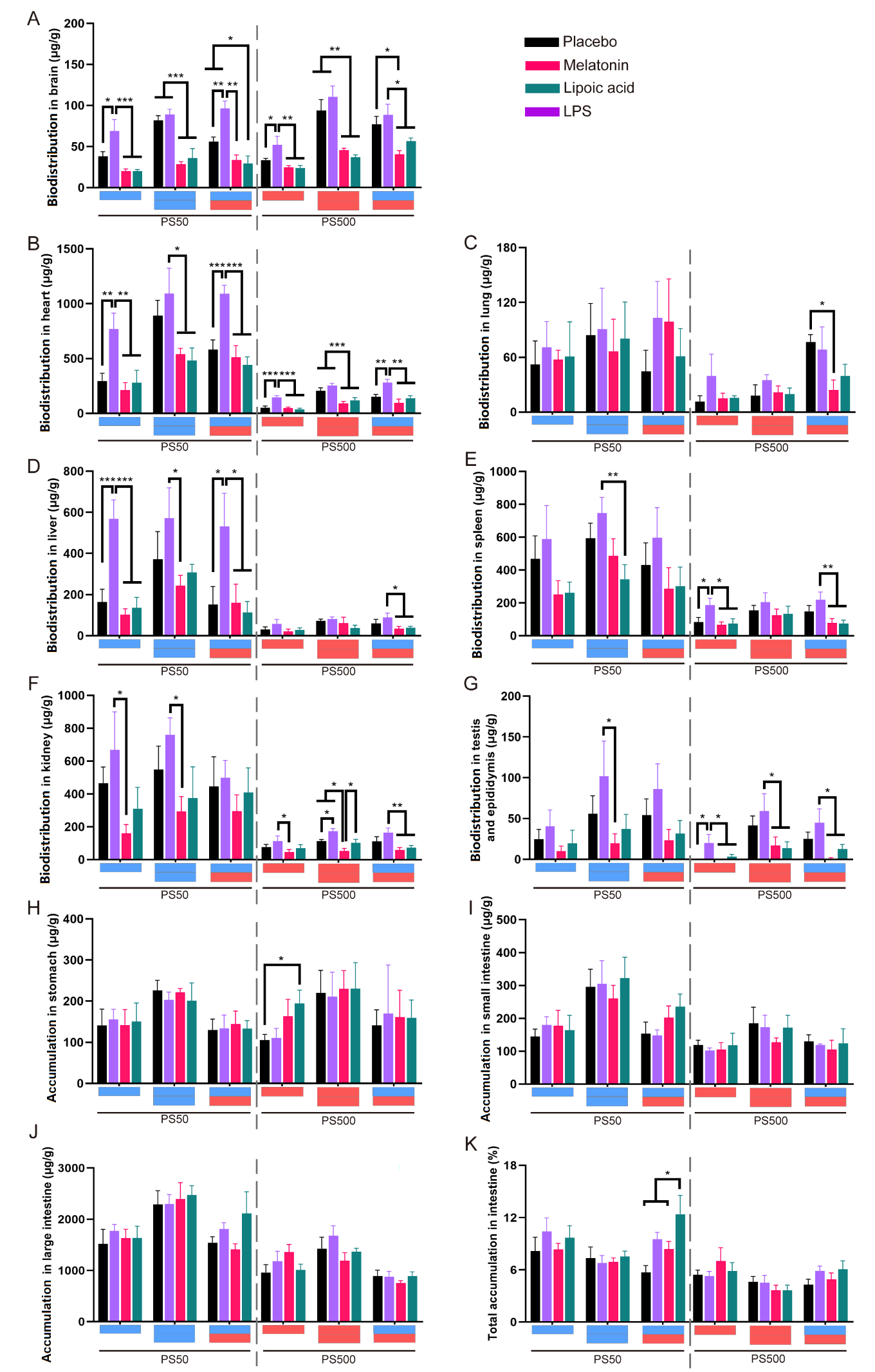
**

**Figure S11.** The organ biodistribution of PS micro- and nanoplastics after oxidant or antioxidant treatment. **P* < 0.05, ***P* < 0.01, and ****P* < 0.001. Results were showed as means ± SE. Comparisons were made with ANOVA, followed by a Tukey’s method (*n* = 5 per group). The color bars were used for grouping, one bar represented a dose of 250 mg/kg body weight, and two bars with different colors represented the mix exposure group with a dose of 250 mg/kg body weight of each particle.

**
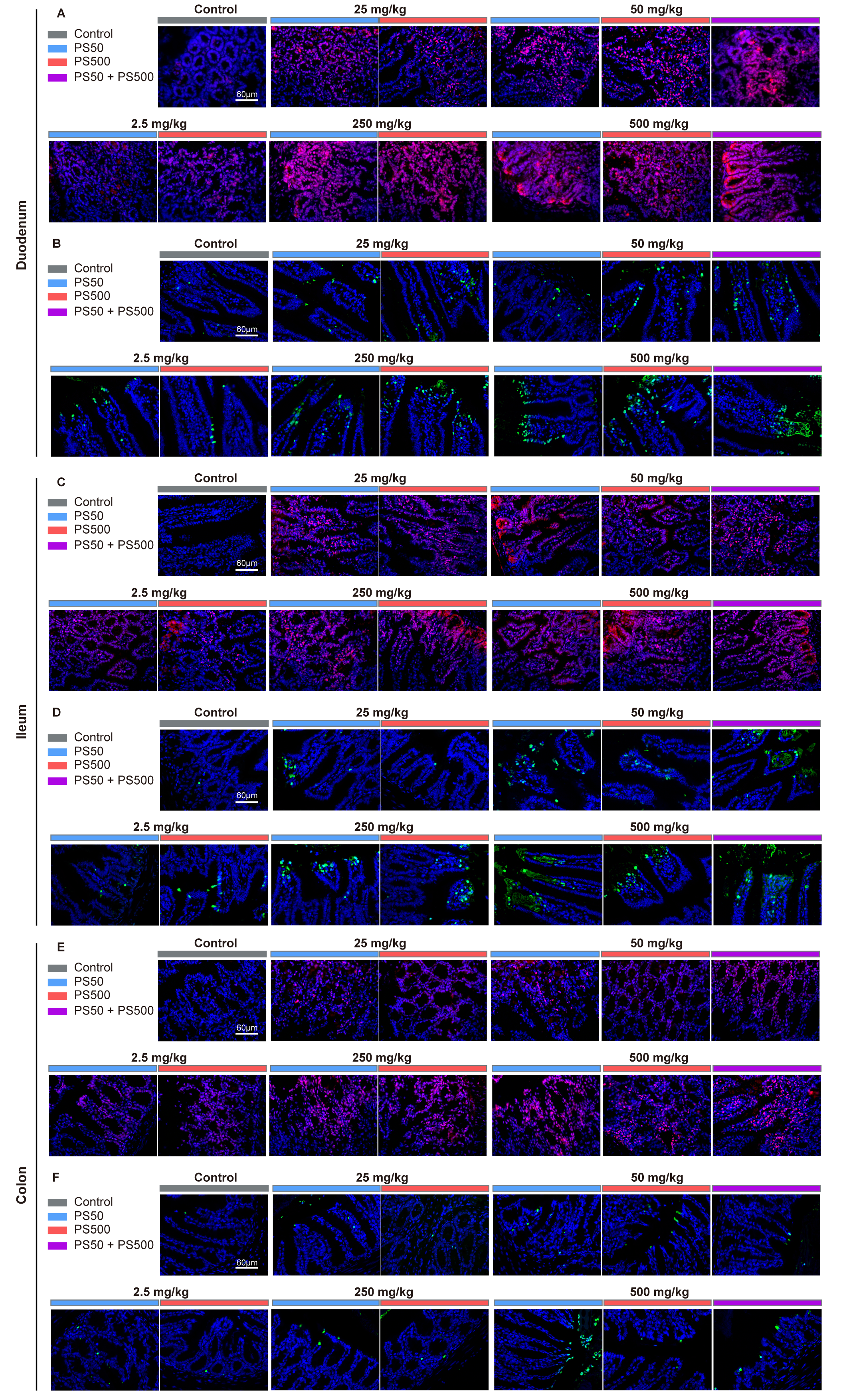
**

**Figure S12.** ROS generation and apoptosis in the duodenum, ileum and colon after exposure for 28 days. A) Representative merged images of the duodenum sections stained with DHE to assess the ROS generation after repeated dose of PS micro- and nanoplastics exposure for 28 days. The ROS was stained in red and the nucleus was stained with DAPI in blue. B) Representative merged images of the duodenum sections stained with TUNEL to assess cell apoptosis after repeated dose of PS micro- and nanoplastics exposure for 28 days. The apoptotic cells were stained in green and the nucleus was stained with DAPI in blue. C) Representative merged images of the ileum sections stained with DHE to assess the ROS generation after repeated dose of PS micro- and nanoplastics exposure for 28 days. The ROS was stained in red and the nucleus was stained with DAPI in blue. D) Representative merged images of the ileum sections stained with TUNEL to assess cell apoptosis after repeated dose of PS micro- and nanoplastics exposure for 28 days. The apoptotic cells were stained in green and the nucleus was stained with DAPI in blue. E) Representative merged images of the colon sections stained with DHE to assess the ROS generation after repeated dose of PS micro- and nanoplastics exposure for 28 days. The ROS was stained in red and the nucleus was stained with DAPI in blue. F) Representative merged images of the colon sections stained with TUNEL to assess cell apoptosis after repeated dose of PS micro- and nanoplastics exposure for 28 days. The apoptotic cells were stained in green and the nucleus was stained with DAPI in blue. The color bars were used for grouping, and the exposure doses were showed by the color bars. *n* = 5 per group.


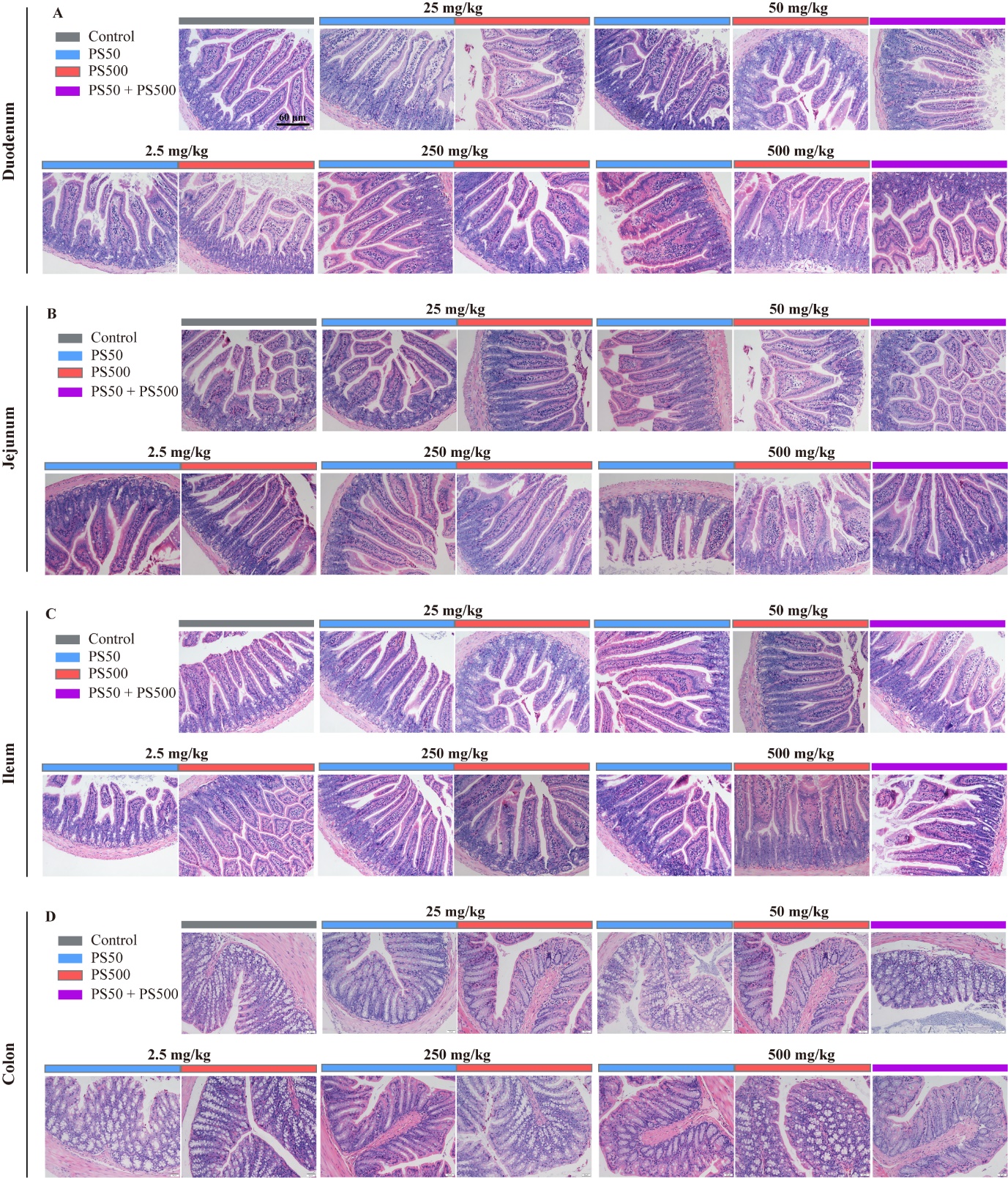


**Figure S13.** H&E staining of the intestinal segments after exposure for 28 days. H&E staining of A) duodenum, B) jejunum, C) ileum and D) colon after 28-day repeated dose of PS micro- and nanoplastics exposure (*n* = 5 per group).

**Table S1.** Physical characteristics of PS micro- and nanoplastics (*n* = 3 per group).

| Medium | Sample | Dynamic Light Scattering | | |
| --- | --- | --- | --- | --- |
|  |  | Size (nm) | PDI | Zeta potential (mV) |
| Distilled water | NF-PS50 | 54.7 ± 0.1 | 0.045 ± 0.013 | -38.3 ± 2.2 |
| Distilled water | NF-PS500 | 516.6 ± 5.2 | 0.054 ± 0.029 | -50.8 ± 1.0 |
| Distilled water | NF-PS50 + NF-PS500 | 59.7 ± 2.7 /  841.2 ± 35.8 | 0.623 ± 0.009 | -46.4 ± 0.8 |
| Gastric Juice | NF-PS50 | 1986.0 ± 263.0 | 0.319 ± 0.085 | -10.9 ± 0.6 |
| Gastric Juice | NF-PS500 | 1156.0 ± 42.7 | 0.307 ± 0.029 | -13.3 ± 0.9 |
| Gastric Juice | NF-PS50 + NF-PS500 | 1907.0 ± 83.8^a^ | 0.317 ± 0.053 | -13.7 ± 0.3 |
| Intestine Juice | NF-PS50 | 121.5 ± 11.0 | 0.900 ± 0.100 | --26.7 ± 1.5 |
| Intestine Juice | NF-PS500 | 613.6 ± 57.6 | 0.293 ± 0.034 | -28.0 ± 2.7 |
| Intestine Juice | NF-PS50 + NF-PS500 | 148.9 ± 45.2 /  1020.2 ± 199.0 | 0.648 ± 0.171 | -25.9 ± 1.1 |
| Distilled water | RF-PS50 | 51.4 ± 1.8 | 0.049 ± 0.011 | -38.4 ± 1.4 |
| Distilled water | RF-PS500 | 507.4 ± 6.8 | 0.057 ± 0.03 | -49.7 ± 0.6 |
| Distilled water | GF-PS50 | 50.2 ± 0.6 | 0.048 ± 0.014 | -38.7 ± 1.7 |
| Distilled water | GF-PS500 | 513.8 ± 5.2 | 0.055 ± 0.027 | -47.5 ± 0.8 |
| Distilled water | RF-PS50 +  GF-PS500 | 53.4 ± 1.7 /  781.3 ± 45.9 | 0.684 ± 0.019 | -49.7 ± 0.8 |
| Gastric Juice | RF-PS50 | 2054.0 ± 215.2 | 0.317 ± 0.099 | -10.4 ± 0.6 |
| Gastric Juice | RF-PS500 | 1226.0 ± 107.6 | 0.303 ± 0.022 | -12.8 ± 1.0 |
| Gastric Juice | GF-PS50 | 2044.2 ± 297.4 | 0.334 ± 0.045 | -10.6 ± 0.7 |
| Gastric Juice | GF-PS500 | 1294.6 ± 94.6 | 0.349 ± 0.040 | -13.0 ± 0.9 |
| Gastric Juice | RF-PS50 +  GF-PS500 | 1857.3 ± 103.3^a^ | 0.327 ± 0.033 | -13.0 ± 0.3 |
| Intestine Juice | RF-PS50 | 98.0 ± 4.2 | 0.511 ± 0.020 | -27.1 ± 1.9 |
| Intestine Juice | RF-PS500 | 638.6 ± 66.4 | 0.318 ± 0.065 | -27.2 ± 2.2 |
| Intestine Juice | GF-PS50 | 116.2 ± 10.0 | 0.515 ± 0.01 | -27.7 ± 1.4 |
| Intestine Juice | GF-PS500 | 634.8 ± 45.8 | 0.311 ± 0.028 | -27.5 ± 3.9 |
| Intestine Juice | RF-PS50 +  GF-PS500 | 151.4 ± 52.2 / 997.2 ± 202.0 | 0.711 ± 0.091 | -24.3 ± 1.1 |

^a^Only one peak of size was detected. NF, non-fluorescence; RF, red fluorescence; GF, green fluorescence.

**Table S2.** Primer sequences for qPCR.

| Gene | Forward Primer (5'-3') | Reverse Primer (5'-3') |
| --- | --- | --- |
| *Muc1* | GGCATTCGGGCTCCTTTCTT | TGGAGTGGTAGTCGATGCTAAG |
| *Muc2* | ATGCCCACCTCCTCAAAGAC | GTAGTTTCCGTTGGAACAGTGAA |
| *Muc3* | CCGAGAGCGGAAGTGTGTG | TGTAACTGTGGTTTTGGTCTTCA |
| *Muc4* | CCTCCTCTTGCTACCTGATGC | GGAACTTGGAGTATCCCTTGTTG |
| *Muc13* | GATCTCTGCAACCCTAACCCC | TCCTTTCACACATGACGACAG |
| *ZO1* | GCCGCTAAGAGCACAGCAA | GCCCTCCTTTTAACACATCAGA |
| *ZO2* | ATGGGAGCAGTACACCGTGA | GCTGAACGGCAAACGAATGG |
| *ZO3* | CTGTGGAGAACGTCACATCTG | CGGGGACGCTTCACTGTAAC |
| *Ocln* | TGAAAGTCCACCTCCTTACAGA | CCGGATAAAAAGAGTACGCTGG |
| *Cldn1* | TGCCCCAGTGGAAGATTTACT | CTTTGCGAAACGCAGGACAT |
| *Cldn2* | CAACTGGTGGGCTACATCCTA | ATCCAGAGGCCCTTGGAAAAG |
| *Cldn3* | ACCAACTGCGTACAAGACGAG | CGGGCACCAACGGGTTATAG |
| *Cldn4* | ATGGCGTCTATGGGACTACAG | GAGCGCACAACTCAGGATG |
| *Cldn5* | GCAAGGTGTATGAATCTGTGCT | GTCAAGGTAACAAAGAGTGCCA |
| *Cldn6* | TGCAAGGTGTATGACTCACTGT | GACGAGACTTGGAGTTCCTATCT |
| *Cldn7* | GGCCTGATAGCGAGCACTG | TGGCGACAAACATGGCTAAGA |
| *Cldn8* | GCAACCTACGCTCTTCAAATGG | TTCCCAGCGGTTCTCAAACAC |
| *Cldn10* | CGGTCAGCCTGGGATTTTTC | GCCTCCGACTTTGGTACATTTC |
| *Cldn11* | GTGGTGGGTTTCGTCACGAG | CGTCCATTTTTCGGCAGGTG |
| *Cldn12* | CTTCCTGTGTGGTATTGCCTC | AAATCGTCAGGTTCTTCTCGTTT |
| *Cldn13* | ATGGTCGTCAGCAAACAAGAG | CATCATCTGGAAAGGTCACCC |
| *Cldn14* | GTCCTGGACCACGAATGACG | GGCCGATTTCAAACTTCATGC |
| *Cldn15* | ATGTCGGTAGCTGTGGAGAC | GGACGGAAAGTCCCAGCAG |
| *Cldn16* | CTGGACAGACTGTTGGATGGT | TATGGAGTCGTACTCATCGCA |
| *Cldn17* | CTTGGATTCTTCGGTTTGGTTGG | CTGCCGATGAAAGCTGACAC |
| *Cldn18* | CTGTACGAGCCCTGATGATCG | CATCCATGCTACCAATGCGAAT |
| *Cldn19* | ATGCAGGCGATGCCATCAT | CCAGGAGTGAATCGTAGAGTTTG |
| *Cldn20* | CTGGGATGTTTAGCTGCACTC | GCAGTTCATTCCTGCTATCGAA |
| *Cldn22* | ATGGGCTTAGTCTTCCGAACG | CTTCCAGTGCGGCAAGTAGTT |
| *Cldn23* | CCCGACGAGTGGAACTACTTC | GGCCAGCGACGAAAAACAC |
| *β-actin* | GGCTGTATTCCCCTCCATCG | CCAGTTGGTAACAATGCCATGT |
| *Ecad* | CAGTTCCGAGGTCTACACCTT | TGAATCGGGAGTCTTCCGAAAA |

**Table S3.** Body weight of 28-day repeated dose oral toxicity study of PS micro- and nanoplastics exposure (*n* = 10 per group) ^a^.

| Particles | Doses (mg/kg body weight) | 0 d  (g) | 7 d  (g) | 14 d  (g) | 21 d  (g) | 28 d  (g) |
| --- | --- | --- | --- | --- | --- | --- |
| Control | 0 | 21.8 ± 1.6 | 22.1 ± 1.8 | 23.3 ± 2.0 | 24.6 ± 2.2 | 24.9 ± 2.2 |
| PS50 | 2.5 | 21.8 ± 0.9 | 22.7 ± 1.5 | 23.8 ± 1.5 | 25.3 ± 1.4 | 25.9 ± 1.7 |
|  | 25 | 21.8 ± 1.6 | 22.7 ± 1.8 | 23.8 ± 2.0 | 25.0 ± 2.5 | 25.8 ± 2.6 |
|  | 50 | 22.2 ± 1.3 | 23.4 ± 1.3 | 24.8 ± 1.5 | 25.9 ± 1.7 | 26.5 ± 1.8 |
|  | 250 | 21.5 ± 1.7 | 22.2 ± 2.0 | 23.0 ± 2.3 | 24.5 ± 2.4 | 25.5 ± 2.5 |
|  | 500 | 21.6 ± 1.5 | 22.7 ± 1.8 | 23.7 ± 2.0 | 24.7 ± 2.2 | 25.2 ± 2.3 |
| PS500 | 0 | 21.4 ± 1.3 | 22.2 ± 1.6 | 22.8 ± 2.1 | 24.8 ± 1.9 | 25.2 ± 1.8 |
|  | 2.5 | 21.5 ± 1.4 | 22.4 ± 1.5 | 23.3 ± 1.6 | 24.7 ± 1.8 | 25.3 ± 1.9 |
|  | 25 | 22.0 ± 1.3 | 23.1 ± 1.4 | 24.1 ± 1.6 | 25.1 ± 2.2 | 26.3 ± 1.5 |
|  | 50 | 22.4 ± 1.5 | 23.5 ± 1.8 | 24.4 ± 2.0 | 25.1 ± 1.9 | 25.2 ± 2.6 |
|  | 250 | 21.3 ± 1.1 | 22.5 ± 1.1 | 23.5 ± 1.3 | 24.7 ± 1.1 | 25.3 ± 1.1 |
|  | 500 | 22.2 ± 1.3 | 23.0 ± 1.6 | 23.8 ± 1.9 | 25.6 ± 1.7 | 26.4 ± 2.0 |
| PS50 + PS500 | 25 + 25 | 22.3 ± 0.9 | 23.1 ± 1.2 | 23.8 ± 1.5 | 25.6 ± 1.8 | 26.0 ± 1.7 |
|  | 250 + 250 | 21.8 ± 1.6 | 22.1 ± 1.8 | 23.3 ± 2.0 | 24.6 ± 2.2 | 24.9 ± 2.2 |

^a^There were not significant changes in the body weight of mice among various groups (*P* > 0.05).
